# Predictive Value of Machine Learning for Poststroke Mortality Risk: Systematic Review and Meta-Analysis

**DOI:** 10.2196/83821

**Published:** 2026-04-02

**Authors:** Yujie Chen, Zhujing Ou, Yiting Deng, Anling Luo, Xuzi Li, Yujia Yang, Qin Yang, Xintong Wu, Muke Zhou

**Affiliations:** 1 Department of Neurology, West China Hospital, Sichuan University Chengdu China

**Keywords:** stroke, artificial intelligence, mortality, prognosis, logistic regression, prediction model

## Abstract

**Background:**

People with stroke face a high mortality risk, and an accurate prediction model is essential to the guidance of clinical decision-making in this population. Recently, with growing attention paid to machine learning (ML) in stroke care, some researchers have investigated the effectiveness of ML in predicting the mortality risk in stroke. However, systematic evidence is still lacking for its effectiveness.

**Objective:**

This systematic review aims to evaluate the value of ML in predicting the stroke mortality risk. The findings are expected to offer an evidence-based basis for developing and assessing clinical risk prediction tools.

**Methods:**

A search was made in Cochrane Library, PubMed, Embase, and Web of Science up to June 23, 2025, and studies that reported a complete performance of ML in predicting stroke mortality were included. Studies with only risk factors analyzed were excluded. The risk of bias of the included studies was assessed using PROBAST (Prediction model Risk of Bias Assessment Tool). Pooled risk ratios with 95% CIs and prediction intervals (PIs) were derived using the Hartung-Knapp-Sidik-Jonkman method under a random-effects model. Subgroup analyses were also conducted by model type, stroke type, patient source, and treatment background. Moreover, a metaregression was conducted on the C-index for out-of-hospital mortality at different time points to explore the influence of time factors on the model’s predictive performance.

**Results:**

Sixty-eight studies were included (23 predicting in-hospital mortality and 45 predicting out-of-hospital mortality), describing the development of 75 prediction models and 43 external validations. The follow-up period was 1 month to 15 years. For predicting in-hospital mortality, the external validation set had a pooled C-index of 0.727 (95% CI 0.677-0.781, 95% PI 0.521-1.000), with sensitivity and specificity of 0.64 (95% CI 0.57-0.70) and 0.74 (95% CI 0.70-0.77), respectively. For predicting out-of-hospital mortality, the pooled C-index was 0.847 (95% CI 0.808-0.887, 95% PI 0.750-0.956) in the external validation set, with sensitivity and specificity of 0.71 (95% CI 0.55-0.82) and 0.76 (95% CI 0.74-0.78), respectively. Comparatively, the overall pooled C-indexes were 0.788 (95% CI 0.766-0.810, 95% PI 0.621-0.999) and 0.812 (95% CI 0.798-0.826, 95% PI 0.693-0.952), respectively. The metaregression revealed a gradual decline in the predictive performance of the overall model and logistic regression model alone, whereas a random forest model maintained sustained performance. Age, National Institutes of Health Stroke Scale score, and stroke-related complications were the most frequently used variables for modeling.

**Conclusions:**

This is the first meta-analysis to demonstrate that ML-based prediction of stroke mortality is feasible. The performance of ML supports its role as an auxiliary tool for identifying high-risk populations, thereby optimizing clinical monitoring and resource allocation. However, due to substantial heterogeneity and a relatively high risk of bias in available studies, caution is warranted in real-world application. The effectiveness of ML may vary across settings, and external validation is recommended before broader implementation.

**Trial Registration:**

PROSPERO CRD420251086321; https://www.crd.york.ac.uk/PROSPERO/view/CRD420251086321

## Introduction

### Rationale

Stroke, an acute focal neurological deficit with cerebrovascular causes, is primarily classified into ischemic stroke and hemorrhagic stroke (intracerebral hemorrhage and subarachnoid hemorrhage) according to its pathogenesis. Acute ischemic stroke (AIS) is the most frequent type, accounting for 60%-80% of the total [1]. As a leading cause of mortality and long-term disability globally, stroke greatly reduces patients’ quality of life and brings a heavy socioeconomic burden [2,3]. Notably, stroke mortality is projected to rise by 50% from 2020 to 2050 [4]. Therefore, stroke has become a serious threat to human health.

The functional prognosis of some patients with ischemic stroke (especially cases within the time window) can benefit from aggressive treatments (eg, intravenous and mechanical thrombolysis). However, a large number of patients are still at risk of adverse outcomes, which deserves adequate attention. The global stroke mortality is high, with approximately 7.5 million deaths in 2021 [5]. In countries with high population density (eg, China), people with AIS have an in-hospital mortality or discharge against medical advice of about 6% [6], a 3-month mortality of 1.5%-3.2%, and a 1-year mortality of 3.4%-6% [7,8]. The 90-day mortality of AIS shows no significant difference between patients undergoing endovascular thrombectomy and those not undergoing endovascular thrombectomy [9]. Moreover, people with intracerebral hemorrhage have an in-hospital mortality or discharge against medical advice of approximately 21.8%, and a 1-year mortality of 17.9% [10]. Notably, unlike highly lethal acute-phase hemorrhagic stroke, ischemic stroke presents a unique pattern of “low acute-phase mortality and high medium- to long-term risks” [11,12], and its mortality is mostly attributed to complications, recurrence, and long-term dysfunction. As a result, time-dynamic risk prediction tools are urgently needed for patients with stroke. Developing stage-specific prediction protocols for high-risk stroke is of great value in early intervention, medical counseling, and resource optimization.

Although numerous studies have sought to predict poststroke functional outcome [13,14], relevant models apply primarily to those patients with rehabilitation potential. However, critically ill intensive care unit patients are faced with a unique and urgent requirement during clinical decision-making, that is, to accurately assess mortality risk to guide physician-patient communication, treatment strategies, and allocation of health care resources [15]. Nowadays, validated prediction tools are still lacking for the stroke mortality risk, and clinical assessment relies on traditional tools (eg, Acute Physiology and Chronic Health Evaluation [APACHE] II score). However, such a single-mode assessment is static and subjective, ignoring the inherent complexity of conditions [16]. Some studies have also been conducted on specific biomarkers. For example, the triglyceride-glucose index (an important biochemical indicator for insulin resistance) can predict AIS mortality [17], and adiponectin is independently associated with post-AIS 5-year mortality [18]. However, early prediction of poststroke death by these prediction tools is still lacking in clinical practice. In addition, the Delphi method can also be applied to the prediction of mortality risk, but the resulting bias is a serious clinical challenge. Therefore, developing early prediction tools for the mortality risk is urgently needed.

### Objectives

As big data and artificial intelligence (AI) emerge, modeling by the growing volume of patient-related data has become a hot spot, which enables individualized outcome prediction [19]. For example, Bonkhoff and Grefkes [20] argued that machine learning (ML) plus clinical, electrophysiological, and neuroimaging data exhibit a high potential for poststroke functional recovery; Akay et al [21] found that AI achieves individualized outcome prediction and assists in treatment decision-making by analyzing multimodal data. The advantage of ML lies in its capability to integrate multisource heterogeneous data from traditional clinical practice, such as medical histories and medical images, and transform them into efficient predictive systems [22]. Unlike traditional methods reliant on rigorous statistical hypotheses, ML presets no data distributions and possesses the capability to process high-dimensional, large-scale data. Therefore, ML demonstrates higher accuracy and efficacy in disease classification and prognosis evaluation [23,24]. Given that patients with stroke often present with complex conditions and multiple complications, and predictions rely heavily on subjective neurological examinations nowadays, it is significant to integrate multimodal clinical data by ML for enhancing the predictive power for stroke mortality [25]. Under this context, some studies have also further investigated ML for predicting short- [26,27] or long-term [28,29] poststroke mortality based on individual data. However, the predictive performance of these models lacks systematic evidence due to the diversity of ML methods and variables, which is a challenge against the subsequent development of simpler clinical prediction tools. No studies have yet quantitatively assessed the effects of these ML models, so the clinical usefulness of the available predictive systems in routine practice remains unclear. Therefore, we conducted this meta-analysis with the following objectives: (1) to measure the predictive performance of ML for stroke mortality across scenarios and summarize the application status of different ML models in stroke mortality prediction, and (2) to evaluate the methodological quality of available studies and determine whether ML can serve as a novel tool for predicting in-hospital and out-of-hospital stroke mortality. The findings are expected to offer an evidence-based basis for facilitating AI development in this field and further developing simple and clinically useful ML-based prediction tools.

## Methods

### Study Registration

This meta-analysis followed PRISMA (Preferred Reporting Items for Systematic Reviews and Meta-Analyses) 2020 Guidelines [30] in Multimedia Appendix 1. The protocol was prospectively registered with PROSPERO (CRD420251086321). To incorporate constructive refinements suggested during the peer-review process, several deviations from the original PROSPERO protocol were implemented to enhance the study’s rigor. These updates primarily include (1) restructuring the inclusion or exclusion criteria based on the PICOS framework; (2) expanding the analytical scope, including the addition of subgroup and sensitivity (SE) analyses; and (3) refining the statistical methodology by adopting the Hartung-Knapp-Sidik-Jonkman (HKSJ) approach. Accordingly, the protocol has been updated to version 2.0 on the PROSPERO platform to ensure full alignment with the final manuscript.

### Eligibility Criteria

The eligibility criteria are as follows (Textbox 1):

**Eligibility criteria for study selection.**

Inclusion criteria

- Populations: patients with stroke, including acute ischemic stroke and unspecified stroke types.
- Model construction: studies using artificial intelligence techniques such as machine learning for automated prediction.
- Outcomes: a complete prediction model for mortality risk.
- Study type: case-control, cohort, nested case-control, or case-cohort studies.
- Language: studies reported in English.

Exclusion criteria

- Populations: only patients with hemorrhagic stroke were included.
- Model construction: only risk factors were analyzed, without constructing a mortality prediction model; validation studies solely assessing established scales.
- Outcomes: any of the following evaluation metrics were lacking: receiver operating characteristic curve, C-index, sensitivity, specificity, accuracy, recall, precision, confusion matrix, *F*_1_-score, or calibration curves.
- Study type: conference abstracts published without peer review, meta-analysis/review/guidelines/letter/case report/protocol.
- Publication year: earlier than 2005.
- Language: non-English language original studies.

### Information Sources and Search Strategy

Literature search was carried out in adherence to PRISMA-S (Preferred Reporting Items for Systematic Reviews and Meta-Analyses literature search extension) guidelines [31]. Cochrane Library (CENTRAL), PubMed (NCBI), Embase (Embase.com), and Web of Science (Clarivate) were thoroughly searched as of June 21, 2025. In addition, manual searches were conducted on references cited in systematic review reports on the same or similar topics. Developed based on the database requirements, the search strategy combined both medical subject headings (“Stroke,” “Machine learning,” “Deep learning,” and “Mortality”) and free-text keywords, as well as Boolean operators. To ensure a comprehensive retrieval, free-text keywords and relevant subordinate terms were identified via PubMed and Embase in addition to primary MeSH or Emtree terms and were further supplemented based on our clinical expertise. Details of the search strategy are available in Multimedia Appendix 2. Duplicate records were removed with EndNote 20 software (Clarivate Analytics). Search parameters were set to include only peer-reviewed papers, with no restrictions on geographical location or publication year, and no published search filters were used. No peer review of the search strategy was conducted.

### Selection Process

All identified studies were imported into EndNote 20 software. All identified studies were imported into EndNote 20 software. After duplicate publications were removed, we used EndNote’s filtering functionality to exclude records based on publication type (eg, reviews, case reports, letters, protocols, animal studies, and meeting abstracts), language (non-English), and publication date (pre-2005). Subsequently, 2 independent investigators (YJC and ZJO) screened the titles and abstracts of the remaining records based on predefined criteria regarding study population (stroke), intervention (machine learning), and outcomes (mortality). Thereafter, a rigorous full-text review was performed to exclude studies lacking model evaluation parameters or those that did not involve the development of original models. The screen was followed by cross-checking. In case of any disagreements, a third investigator (MKZ) was consulted for resolution.

### Data Collection Process and Data Items

A spreadsheet was created to extract data, including title, first author, type of study, year of publication, patient source, dataset source, stroke type, treatment background (whether to undergo acute-phase vascular recanalization), follow-up time, total number of death cases in the training set, overfitting methods, number of cases in the validation set, handling methods for missing value, variable selection, model types, and modeling variables. During data extraction, studies with missing outcome data were excluded directly from the meta-analysis. Three independent investigators (YJC, YTD, and ALL) implemented the data extraction, followed by cross-checking. In case of any disagreements, a third investigator (XTW) was consulted for resolution.

### Risk of Bias Assessment

The included studies were assessed for risk of bias (RoB) using the PROBAST (Prediction model Risk of Bias Assessment Tool) [32] by structured questions across 4 domains: participants (2 questions), predictors (3 questions), outcomes (6 questions), and analysis (9 questions). Each question was answered as “Yes,” “Probably yes,” “No,” “Probably no,” or “No information.” Each domain was rated as high RoB if 1 or more questions were answered as “No” or “Probably no,” as low RoB if all answers were “Yes” or “Probably yes,” and as unclear RoB if answered “No information.” The overall risk was deemed low only when all domains were rated as low RoB, whereas any high RoB was considered overall high risk. In the absence of high-risk domains, the overall risk was unclear when 1 or more domains had unclear RoB. Two investigators (YJC and YJY) independently performed RoB assessment and cross-checking. Discrepancies were settled by third-party adjudication.

### Synthesis Methods

The C-index, a measure for the overall predictive accuracy of ML models, underwent a meta-analysis. If the C-index was reported without its 95% CI or standard error, the SE was estimated using the method described by Debray et al [33]. Similarly, SE and specificity (SP) were synthesized by a bivariate mixed-effects model. Meta-analyses of these metrics typically rely on diagnostic 4-fold tables, but most primary studies included did not report such tables. In this case, we sought to reconstruct the diagnostic 4-fold table by leveraging other available performance metrics, such as SE, SP, precision, accuracy, and the total number of events (deaths). Some studies were based on the same database, but significant heterogeneity was present across these studies in the data extraction period, variable selection, model construction, and parameter adjustment rule. Therefore, all eligible studies were included, and given this potential heterogeneity, a random-effects model was adopted for meta-analysis [34]. Multiple entries from the same study represented distinct prediction models or validation methods, which were treated as independent units of evidence [35]. We performed random-effects meta-analyses using the HKSJ method to estimate pooled effect sizes with 95% CIs. The HKSJ method was chosen as it provides more robust inference, particularly when the number of studies is small. In addition, we calculated 95% prediction intervals (PIs) to present the expected range of true effects in similar studies, across clinical settings, or in future studies, and to better express the extent of heterogeneity [36,37]. The width of PIs was directly influenced by the number of included studies and the extent of heterogeneity [38]. Model performance was evaluated separately in training and validation sets. Furthermore, the performance of models for in-hospital and out-of-hospital mortality was separately synthesized.

To explore potential sources of heterogeneity, subgroup analyses were conducted, stratified by stroke type (AIS vs not specific), patient source (public database vs local hospital), treatment background (reperfusion therapy vs not specific), and model type. Furthermore, to evaluate the generalizability of the models and minimize optimistic bias associated with internal validation [39], we conducted a subgroup analysis in the validation set by validation method (internal validation vs external validation). When reporting the results, we prioritized the performance of the external validation subgroup based on the primary importance of external validation results for assessing clinical generalization [35]. The overall pooled performance (combining internal and external validation) was subsequently reported as a reference during development setting. Given the limited number of external validation studies, subgroup analyses were based on the overall performance. We used a random-effects model to calculate pooled estimates within each subgroup and a fixed-effects (plural) model to compare effect sizes across subgroups. A random-effects metaregression was conducted to investigate the association between the C-index of model performance and the follow-up time after discharge in studies reporting out-of-hospital mortality. The small-study effect in the validation sets of included studies and different models (≥10 studies) in the validation sets was qualitatively and quantitatively assessed with funnel plots and Egger regression tests, respectively. Funnel plot asymmetry was visualized to detect the small-study effect.

SE analyses were performed in 2 stages. In the first stage, to assess the potential impact of patient cohort overlap from using the same public databases (Medical Information Mart for Intensive Care [MIMIC-III/IV], eICU Collaborative Research Database [eICU-CRD], and Sentinel Stroke National Audit Programme [SSNAP]) on the pooled results, only 1 study with a large sample size, high methodological quality (based on PROBAST), and complete analysis (using external validation) was retained per public database. All studies from single-center or independent regional registries were also retained. Using independent datasets filtered in the 2-stage ways, a meta-analysis of the C-index for in-hospital and out-of-hospital mortality prediction models was conducted.

To mitigate potential bias arising from studies reporting multiple models, we established a priority rule for the second stage: selecting only the best-performing model from each study. Specifically, for studies with multiple models, we prioritized the optimal model among those with external validation; if no external validation was performed, the model with the highest internal performance was selected. In this way, we could obtain the evidence of the model with the greatest potential for clinical generalization or optimal performance from each study of independent sets, thereby assessing the robustness of the main findings under this stricter criterion. Additionally, following this criterion (the principle of selecting the study with the largest sample size among overlapping validation sets and subsequently selecting the optimal model within each independent validation set), a separate meta-analysis of the C-index was conducted in the external validation set. It should be noted that the SE analyses were performed post hoc, and thus they were not prespecified in the preregistered study protocol or initial data analysis plan. Statistical analyses and visualizations were performed using Stata (version 15.0; StataCorp, LLC) and R (version 4.5.1; R Foundation for Statistical Computing), using metafor, forestplot, and ggplot2 packages.

### Protocol Deviations and Updates

To incorporate constructive refinements from the peer-review process, several deviations from the original PROSPERO protocol were implemented to enhance the study’s rigor. These updates primarily include (1) restructuring the inclusion or exclusion criteria according to the PICOS framework, (2) refining the statistical methodology by adopting the HKSJ method and reporting PIs, and (3) expanding the scope of analysis by adding SE and subgroup analyses (eg, internal vs external validation). Accordingly, the study protocol has been updated to version 2.0 on the PROSPERO platform to ensure full alignment with the final paper.

## Results

### Study Selection

**Figure 1:**
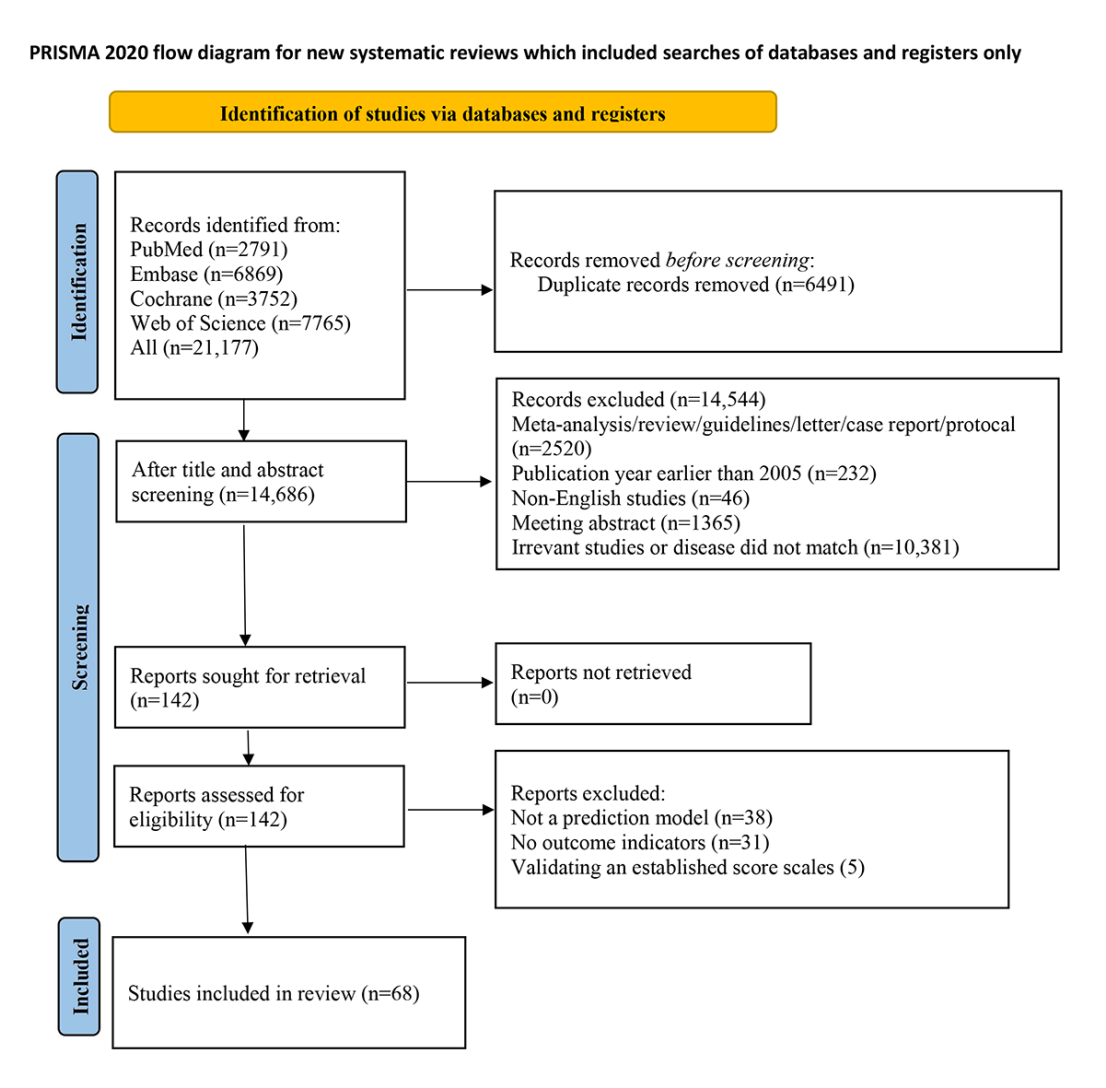
PRISMA (Preferred Reporting Items for Systematic Reviews and Meta-Analyses) flowchart.

We initially retrieved 21,177 records, of which 6491 duplicate publications were eliminated. After title and abstract review, 14,544 studies were further excluded. Then the full text of the remainder was examined, of which 38 were excluded due to no modeling, 31 due to no definite outcome metrics, and 5 due to a focus only on validation of previous scores. Finally, 68 studies were included [26-29,40-103] (Figure 1).

### Study Characteristics

We included 68 studies published between 2005 and 2025, mainly from 2007 to 2025. They came from 19 countries and involved 1,759,472 participants, of whom 225,598 (12.8%) died. Twenty-three studies predicted the in-hospital mortality risk, and 45 studies predicted the out-of-hospital mortality risk. Three studies reported both in-hospital and out-of-hospital mortality [41,67,100]. Out-of-hospital mortality was followed up for 1-12 months, as well as 2 years [67], 3 years [46,72], 5 years [82], 10 years [45,75], and 15 years [40] in a few studies. Public databases, including MIMIC, eICU-CRD, the Minimum Basic Data Set, and SSNAP, were used in 28 studies, and there were 26 single-center studies and 14 multicenter studies. Forty-two studies focused on AIS, and the remaining 26 did not specify the stroke type. Seven studies predicted the mortality risk following acute-phase vascular recanalization. Logistic regression (LR) was the most frequently used model (51/68, 75%), followed by extreme gradient boosting (XGBoost) (21/68, 31%), random forest (RF) (20/68, 29%), support vector machine (11/68, 16%), and deep learning (DL) (2/68, 3%). For modeling variables, radiomics features were adopted in only 2 studies, and clinical features were adopted in the remainder. The generation method for the validation set was clearly described in 64 studies. Thirteen studies performed independent external validation, and 11 studies adopted both internal and external validation. For internal validation, random sampling was used in 44 studies, Bootstrap in 9 studies, k-fold cross-validation in 6 studies, holdout validation in 2 studies, and temporal validation in 1 study (Table S1 in Multimedia Appendix 2).

### RoB in Studies

For participant selection, PROBAST considers retrospective cohort or case-control studies that do not use public databases with high RoB. Since 8 case-control studies did not use publicly available databases, they were rated as high RoB. All included studies developed reasonable eligibility criteria, so no RoB was introduced in this dimension.

The predictors had the same definition and assessment among participants in all studies, and they were all valid, so all studies were rated as low RoB. The outcome event was death, and the possibility of assessment with knowledge of the outcome could not be ruled out for case-control studies, so we rated 8 studies as high RoB.

The outcome in this meta-analysis was death or nondeath, and it was reasonably categorized and defined. The definition of the outcome eliminated the influence of predictors and was consistent among participants, and the outcome was assessed objectively with or without knowledge of the predictor. Therefore, all studies were rated as low RoB in this dimension.

For statistical analysis, 11 studies were assessed as high RoB because of inappropriate sample sizes; 11 as high RoB because subjects with missing data were directly excluded, without appropriate handling, and 3 as unclear RoB due to no description of the handling method for missing data. The RoB in 13 studies was high since only univariate analysis was conducted. The RoB in 10 studies was high for failing to account for data complexity, including high dimensionality, multicollinearity, or unbalanced data handling. Three studies were rated as high RoB for inappropriate internal validation. All studies were rated as low RoB since they all appropriately handled and incorporated continuous or categorical independent variables and assessed model performance.

To sum up, the overall RoB was low in 33 studies and high in 35 studies (Table S2 in Multimedia Appendix 2 [26-29,40-103] and Multimedia Appendix 3).

### Results of Synthesis

#### In-hospital Mortality

**Figure 2:**
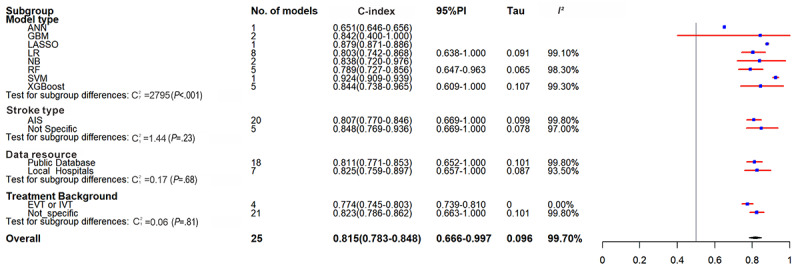
Forest plot for meta-analysis of C-index of predictive models for in-hospital mortality in stroke in the training set. AIS: acute ischemic stroke; ANN: artificial neural network; EVT: endovascular thrombectomy; GBM: gradient boosting machine; IVT: intravenous thrombolysis; LASSO: least absolute shrinkage and selection operator; LR: logistic regression; NB: Naive Bayes; PI: prediction interval; RF: random forest; SVM: support vector machine; XGBoost: extreme gradient boosting.

Twenty-five ML models for predicting in-hospital mortality were extracted and included 1,345,411 patients, of whom 159,409 (11.7%) died. The pooled C-index was 0.815 (95% CI 0.783-0.848, 95% PI 0.666-0.997) (Table 1 and Figure 2). The C-index was 0.807 (95% CI 0.770-0.846, 95% PI 0.669-1.000) in studies on AIS, 0.811 (95% CI 0.771-0.853, 95% PI 0.652-1.000) in studies based on public databases, 0.825 (95% CI 0.759-0.897, 95% PI 0.657-1.000) in studies based on hospital databases, and 0.774 (95% CI 0.745-0.803, 95% PI 0.739-0.810) in studies explicitly reporting acute-phase vascular recanalization. LR, RF, and XGBoost models were dominated, and the LR model had a C-index of 0.803 (95% CI 0.742-0.868, 95% PI 0.638-1.000).

In addition, diagnostic metrics could be calculated in 24 studies in the training set, with a pooled SE of 0.81 (95% CI 0.70-0.88) and SP of 0.82 (95% CI 0.76-0.87). The SE and SP of the LR model were 0.71 (95% CI 0.61-0.79) and 0.78 (95% CI 0.69-0.85) (Table 2 and Figures S1 and S2 in Multimedia Appendix 2).

**Table 1:** Results of meta-analysis of C-index of machine learning for predicting in-hospital mortality in stroke.

Subgroup analysis			Training set													Validation set										
			n		Sample size		C-index (95% CI)		95% PI^a^		Tau		*I*^2^ (%)		n			Sample size		C-index (95% CI)		95% PI		Tau		*I*^2^ (%)
**Model**																										
	ANN^b^	1		14,8891		0.651 (0.646-0.656)		N/A^c^		N/A		N/A		8			6837		0.794 (0.696-0.905)		0.544-1.000		0.151		98.40	
	DT^d^	—^e^		—		—		—		—		—		5			5233		0.720 (0.648-0.800)		0.581-0.892		0.068		73.00	
	EL^f^	—		—		—		—		—		—		2			845		0.864 (0.373-1.000)		N/A		N/A		N/A	
	GBM^g^	2		2633		0.842 (0.400-1.000)		N/A		N/A		N/A		2			3813		0.813 (0.248-1.000)		N/A		N/A		N/A	
	KNN^h^	—		—		—		—		—		—		9			10,555		0.756 (0.707-0.805)		0.590-0.967		0.101		92.80	
	LASSO^i^	1		77,653		0.879 (0.871-0.886)		N/A		N/A		N/A		1			9141		0.884 (0.863-0.905)		N/A		N/A		N/A	
	LightGBM^j^	—		—		—		—		—		—		4			2223		0.828 (0.685-1.000)		0.549-1.000		0.114		92.80	
	LR^k^	8		17,2088		0.803 (0.742-0.868)		0.638-1.000		0.091		99.10		26			419,933		0.802 (0.762-0.844)		0.631-1.000		0.114		99.80	
	NB^l^	2		1300		0.838 (0.720-0.976)		N/A		N/A		N/A		5			2464		0.621 (0.339-1.139)		0.146-1.000		0.475		95.40	
	RF^m^	5		15,8453		0.789 (0.727-0.856)		0.647-0.963		0.065		98.30		14			13,721		0.811 (0.773-0.851)		0.682-0.963		0.077		93.20	
	SVM^n^	1		2031		0.924 (0.909-0.939)		N/A		N/A		N/A		7			7056		0.763 (0.715-0.814)		0.643-0.906		0.064		90.50	
	XGBoost^o^	5		12,128		0.844 (0.738-0.965)		0.609-1.000		0.107		99.30		16			269,990		0.803 (0.761-0.847)		0.650-0.991		0.096		97.60	
**Stroke type**																										
	AIS^p^	20		56,6742		0.807 (0.770-0.846)		0.669-1.000		0.099		99.80		65			108,059		0.788 (0.766-0.810)		0.642-0.965		0.101		99.60	
	Not specific	5		8435		0.848 (0.769-0.936)		0.669-1.000		0.078		97.00		34			643,752		0.784 (0.733-0.840)		0.551-1.000		0.171		97.20	
**Data resource**																										
	Public database	18		561,389		0.811 (0.771-0.853)		0.652-1.000		0.101		99.80		87			723,433		0.775 (0.753-0.799)		0.613-0.980		0.117		99.50	
	Local hospital	7		13,788		0.825 (0.759-0.897)		0.657-1.000		0.087		93.50		12			28,378		0.879 (0.840-0.921)		0.749-1.000		0.070		95.70	
**Treatment background**																										
	EVT^q^/IVT^r^	4		572,769		0.774 (0.745-0.803)		0.739-0.810		0.000		0.00		—			—		—		—		—		—	
	Not specific	21		2408		0.823 (0.786-0.862)		0.663-1.000		0.101		99.80		—			—		—		—		—		—	
**Validation method**																										
	Internal validation	—		—		—		—		—		—		67			480,774		0.815 (0.796-0.835)		0.681-0.976		0.089		99.60	
	External validation	—		—		—		—		—		—		32			271 037		0.727 (0.677-0.781)		0.521-1.000		0.170		96.20	
Overall			25		575,177		0.815 (0.783-0.848)		0.666-0.997		0.096		99.70		99			751,811		0.788 (0.766-0.810)		0.621-0.999		0.119		99.50

^a^PI: prediction interval.

^b^ANN: artificial neural network.

^c^N/A: not applicable.

^d^DT: decision tree.

^e^Not available.

^f^EL: ensemble learning.

^g^GBM: gradient boosting machine.

^h^KNN: k-nearest neighbor.

^i^LASSO: least absolute shrinkage and selection operator.

^j^LightGBM: light gradient boosting machine.

^k^LR: logistic regression.

^l^NB: Naive Bayes.

^m^RF: random forest.

^n^SVM: support vector machine.

^o^XGBoost: extreme gradient boosting.

^p^AIS: acute ischemic stroke.

^q^EVT: endovascular thrombectomy.

^r^IVT: intravenous thrombolysis.

**Table 2:** Results of meta-analysis of sensitivity and specificity of machine learning for predicting in-hospital mortality in stroke.

Subgroup analysis			Training set									Validation set						
			n		Sample size		SE^a^ (95% CI)		SP^b^ (95% CI)		n			Sample size		SE (95% CI)		SP (95% CI)
**Model**																		
	ANN^c^	2		14,9231		0.92 (0.86-0.98)		0.81 (0.64-0.97)		9			7177		0.74 (0.58-0.85)		0.79 (0.72-0.85)	
	DT^d^	—^e^		—		—		—		5			5233		0.65 (0.44-0.81)		0.78 (0.52-0.92)	
	EL^f^	—		—		—		—		2			845		0.88 (0.82-0.94)		0.89 (0.86-0.91)	
	GBM^g^	2		2633		0.88 (0.72-0.79)		0.79 (0.75-0.83)		2			3813		0.58 (0.33-0.83)		0.78 (0.58-0.98)	
	KNN^h^	—		—		—		—		9			10,555		0.52 (0.34-0.69)		0.83 (0.70-0.91)	
	LASSO^i^	1		77,653		0.81		0.81		1			9141		0.79		0.84	
	LightGBM^j^	—		—		—		—		4			2223		0.73 (0.58-0.83)		0.82 (0.71-0.90)	
	LR^k^	7		166,373		0.71 (0.61-0.79)		0.78 (0.69-0.85)		19			189,436		0.72 (0.66-0.78)		0.78 (0.70-0.84)	
	NB^l^	2		1300		0.82 (0.70-0.93)		0.74 (0.69-0.77)		5			2464		0.64 (0.36-0.85)		0.70 (0.61-0.77)	
	RF^m^	4		152,738		0.66 (0.62-0.70)		0.78 (0.68,0.86)		13			12,292		0.74 (0.63-0.82)		0.81 (0.69-0.89)	
	SVM^n^	1		2031		0.90		0.85		7			7056		0.61 (0.51-0.70)		0.77 (0.71-0.82)	
	XGBoost^o^	5		6753		0.78 (0.62-0.88)		0.90 (0.77-0.96)		13			138,201		0.69 (0.62-0.75)		0.73 (0.68-0.78)	
**Stroke type**																		
	AIS^p^	17		549,597		0.73 (0.66-0.79)		0.82 (0.75-0.88)		58			102,373		0.70 (0.66-0.73)		0.77 (0.74-0.80)	
	Not specific	7		9115		0.92 (0.67-0.98)		0.83 (0.71-0.90)		31			286,063		0.67 (0.57-0.75)		0.82 (0.74-0.88)	
**Data resource**																		
	Public database	15		544,244		0.74 (0.67-0.80)		0.82 (0.75-0.88)		76			359,666		0.70 (0.66-0.73)		0.75 (0.72-0.77)	
	Local hospital	9		14,468		0.89 (0.67-0.97)		0.82 (0.72-0.89)		13			28,770		0.63 (0.43-0.80)		0.93 (0.87-0.97)	
**Treatment background**																		
	EVT^q^/IVT^r^	4		2408		0.68 (0.64-0.72)		0.76 (0.69-0.82)		N/A^s^			N/A		N/A		N/A	
	Not specific	20		556,304		0.83 (0.71-0.91)		0.83 (0.77-0.88)		N/A			N/A		N/A		N/A	
**Validation method**																		
	Internal validation	—		—		—		—		56			111,5921		0.71 (0.66-0.76)		0.81 (0.77-0.85)	
	External validation	—		—		—		—		33			272,515		0.64 (0.57-0.70)		0.74 (0.70-0.77)	
Overall			24		558,712		0.81 (0.70-0.88)		0.82 (0.76-0.87)		89			388,436		0.69 (0.65-0.73)		0.79 (0.76-0.82)

^a^SE: sensitivity.

^b^SP: specificity.

^c^ANN: artificial neural network.

^d^DT: decision tree

^e^Not available.

^f^EL: ensemble learning.

^g^GBM: gradient boosting machine.

^h^KNN: k-nearest neighbor.

^i^LASSO: least absolute shrinkage and selection operator.

^j^LightGBM: light gradient boosting machine.

^k^LR: logistic regression.

^l^NB: Naive Bayes.

^m^RF: random forest.

^n^SVM: support vector machine.

^o^XGBoost: extreme gradient boosting.

^p^AIS: acute ischemic stroke.

^q^EVT: endovascular thrombectomy.

^r^IVT: intravenous thrombolysis.

^s^N/A: not applicable.

**Figure 3:**
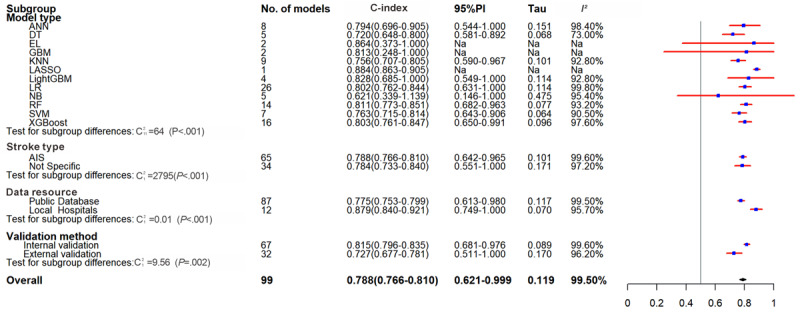
Forest plot for meta-analysis of C-index of predictive models for inhospital mortality in stroke in the validation set. AIS: acute ischemic stroke; ANN: artificial neural network; DT: decision tree; EL: ensemble learning; GBM: gradient boosting machine; KNN: k-nearest neighbor; LASSO: least absolute shrinkage and selection operator; LightGBM: light gradient boosting machine; LR: logistic regression; NA: Not applicable; NA:Not applicable; NB: Naive Bayes; PI: prediction interval; RF: random forest; SVM: support vector machine; XGBoost: extreme gradient boosting.

Based on externally validated models, the analysis indicated a moderate level of generalizability, with a pooled C-index of 0.727 (95% CI 0.677-0.781, 95% PI 0.521-1.000), with an SE of 0.64 (95% CI 0.57-0.70) and an SP of 0.74 (95% CI 0.70-0.77). These performance estimates were lower than the overall pooled results which yielded a C-index of 0.788 (95% CI 0.766-0.810, 95% PI 0.621-0.999), an overall SE of 0.69 (95% CI 0.65-0.73), and an SP of 0.79 (95% CI 0.76-0.82). The models using hospital databases outperformed those using public databases; the former had a C-index of 0.879 (95% CI 0.840-0.921, 95% PI 0.621-0.999), with SP and SE of 0.93 (95% CI 0.87-0.97) and 0.63 (95% CI 0.43-0.80), and the latter had a C-index of 0.775 (95% CI 0.753-0.799, 95% PI 0.613-0.980), with SP and SE of 0.75 (95% CI 0.72-0.77) and 0.70 (95% CI 0.66-0.73). The studies on AIS had a C-index of 0.788 (95% CI 0.766-0.810, 95% PI 0.642-0.965), with SP and SE of 0.70 (95% CI 0.66-0.73) and 0.77 (95% CI 0.74-0.80), and the LR model had a C-index of 0.802 (95% CI 0.762-0.844, 95% PI 0.631-1.000), with SE and SP of 0.72 (95% CI 0.66-0.78) and 0.78 (95% CI 0.70-0.84), consistent with the overall results. The internal validation subgroup aligned with the overall trend but yielded more optimistic estimates, with a pooled C-index of 0.815 (95% CI 0.796-0.835, 95% PI 0.681-0.976), an SE of 0.71 (95% CI 0.66-0.76), and an SP of 0.81 (95% CI 0.77-0.85) (Tables 1 and 2, Figure 3, and Figures S3 and S4 in Multimedia Appendix 2).

### Out-of-Hospital Mortality

**Figure 4:**
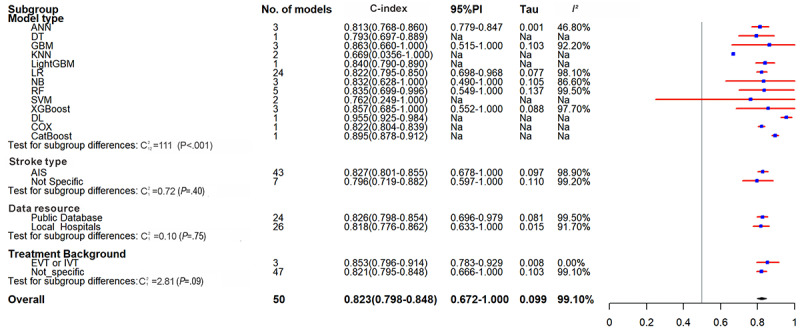
Forest plot for meta-analysis of C-index of predictive models for out-of-hospital mortality in stroke in the training set. AIS: acute ischemic stroke; ANN: artificial neural network; CatBoost: categorical boosting; COX: Cox proportional hazards; DL: deep learning; DT: decision tree; EVT: endovascular thrombectomy; GBM: gradient boosting machine; IVT: intravenous thrombolysis; KNN: k-nearest neighbor; LightGBM: light gradient boosting machine; LR: logistic regression; NA: Not applicable; NB: Naive Bayes; PI: prediction interval; RF: random forest; SVM: support vector machine; XGBoost: extreme gradient boosting.

Fifty ML models for predicting out-of-hospital mortality were extracted and included 414,061 patients, of whom 66,189 (16%) died. The pooled C-index was 0.823 (95% CI 0.798-0.848, 95% PI 0.672-1.000) (Table 3 and Figure 4), and the diagnostic SE and SP were 0.66 (95% CI 0.60-0.72) and 0.88 (95% CI 0.83-0.91) (Figures S5 and S6 in Multimedia Appendix 2).

**Table 3:** Results of meta-analysis of C-index of machine learning for predicting out-of-hospital mortality in stroke.

Subgroup analysis		Training set							Validation set						
		n	Sample size	C-index (95% CI)	95% PI^a^	Tau	*I*^2^ (%)	n		Sample size	C-index (95% CI)	95% PI	Tau	*I*^2^ (%)	
**Model**															
	ANN^b^	3	4330	0.813 (0.768-0.860)	0.779-0.847	0.001	46.80	4		2615	0.822 (0.653-1.000)	0.498-1.000	0.140	95.3	
	DT^c^	1	474	0.793 (0.697-0.889)	N/A^d^	N/A	N/A	3		1906	0.69 5 (0.585-0.825)	0.556-0.868	0.025	0.0	
	EL^e^	—^f^	—	—	—	—	—	3		17,053	0.857 (0.708-1.000)	0.588-1.000	0.075	97.4	
	GBM^g^	3	3333	0.863 (0.660-1.000)	0.515-1.000	0.103	92.20	5		2076	0.788 (0.717-0.866)	0.652-0.953	0.058	57.6	
	KNN^h^	2	2859	0.669 (0.0356-1.000)	N/A	N/A	N/A	4		5205	0.737 (0.637-0.852)	0.578-0.939	0.065	80.1	
	LASSO^i^	—	—	—	—	—	—	—		—	—	—	—	—	
	LightGBM^j^	1	1388	0.840 (0.790-0.890)	N/A	N/A	N/A	2		2116	0.926 (0.665-1.000)	N/A	N/A	N/A	
	LR^k^	24	151,284	0.822 (0.795-0.850)	0.698-0.968	0.077	98.10	27		149,308	0.817 (0.793-0.841)	0.708-0.942	0.068	96.8	
	NB^l^	3	3333	0.832 (0.628-1.000)	0.490-1.000	0.105	86.60	6		6687	0.809 (0.741-0.883)	0.651-1.000	0.076	86.3	
	RF^m^	5	9502	0.835 (0.699-0.996)	0.549-1.000	0.137	99.50	16		17,729	0.833 (0.801-0.865)	0.724-0.957	0.063	83.1	
	RSF^n^	—	—	—	—	—	—	2		383	0.789 (0.630- 0.986)	N/A	N/A	N/A	
	SVM^o^	2	2859	0.762 (0.249-1.000)	N/A	N/A	N/A	7		7225	0.789 (0.721-0.863)	0.620-1.000	0.091	92.1	
	XGBoost^p^	3	7660	0.857 (0.685-1.000)	0.552-1.000	0.088	97.70	10		11,327	0.800 (0.747-0.856)	0.643-0.994	0.091	96.8	
	DL^q^	1	1546	0.955 (0.925-0.984)	N/A	N/A	N/A	2		1161	0.873 (0.799-0.955)	N/A	N/A	N/A	
	COX^r^	1	425	0.822 (0.804-0.839)	N/A	N/A	N/A	5		4991	0.792 (0.720-0.871)	0.632-0.993	0.074	92.2	
	CatBoost^s^	1	1471	0.895 (0.878-0.912)	N/A	N/A	N/A	5		3902	0.818 (0.715-0.937)	0.599-1.000	0.102	94.1	
	AdaBoost^t^	—	—	—	—	—	—	1		1968	0.850 (0.811-0.889)	N/A	N/A	N/A	
**Stroke type**															
	AIS^u^	43	176,770	0.827 (0.801-0.855)	0.678-1.000	0.097	98.90	66		163,990	0.796 (0.778-0.813)	0.677-0.935	0.080	91.4	
	Not specific	7	13,694	0.796 (0.719-0.882)	0.597-1.000	0.110	99.20	36		71,662	0.841 (0.819-0.862)	0.733-0.964	0.066	97.8	
**Data resource**															
	Public database	24	171,811	0.826 (0.798-0.854)	0.696-0.979	0.081	99.50	30		161,344	0.789 (0.763-0.815)	0.661-0.941	0.085	99.4	
	Local hospital	26	18,653	0.818 (0.776-0.862)	0.633-1.000	0.015	91.70	72		74,308	0.824 (0.808-0.841)	0.712-0.955	0.073	87.1	
**Treatment background**															
	EVT^v^/IVT^w^	3	920	0.853 (0.796-0.914)	0.783-0.929	0.008	0.00	7		1338	0.817 (0.760-0.879)	0.681-0.981	0.068	77.5	
	Not specific	47	189,544	0.821 (0.795-0.848)	0.666-1.000	0.103	99.10	95		234,314	0.811 (0.797-0.826)	0.691-0.953	0.081	98.4	
**Validation method**															
	Internal validation	—	—	—	—	—	—	92		230,828	0.808 (0.793-0.823)	0.687-0.950	0.081	98.5	
	External validation	—	—	—	—	—	—	10		4824	0.847 (0.808-0.887)	0.750-0.956	0.050	71.3	
Overall		50	190,464	0.823 (0.798-0.848)	0.672-1.000	0.099	99.10	102		235,652	0.812 (0.798-0.826)	0.693-0.952	0.080	98.3	

^a^PI: prediction interval.

^b^ANN: artificial neural network.

^c^DT: decision tree.

^d^Not applicable.

^e^EL: ensemble learning.

^f^Not available.

^g^GBM: gradient boosting machine.

^h^KNN: k-nearest neighbors.

^i^LASSO: least absolute shrinkage and selection operator.

^j^LightGBM: light gradient boosting machine.

^k^LR: logistic regression.

^l^NB: Naive Bayes.

^m^RF: random forest.

^n^RSF: random survival forest.

^o^SVM: support vector machine.

^p^XGBoost: extreme gradient boosting.

^q^DL: deep learning.

^r^COX: Cox proportional hazards.

^s^CatBoost: categorical boosting.

^t^AdaBoost: adaptive boosting.

^u^AIS: acute ischemic stroke.

^v^EVT: endovascular thrombectomy.

^w^IVT: intravenous thrombolysis.

The pooled C-index was 0.827 (95% CI 0.801-0.855, 95% PI 0.678-1.000) in studies on AIS, 0.826 (95% CI 0.798-0.854, 95% PI 0.696-0.979) in studies based on public databases, 0.818 (95% CI 0.776-0.862, 95% PI 0.633-1.000) in studies based on hospital databases, and 0.853 (95% CI 0.796-0.914, 95% PI 0.783-0.929) in studies reporting acute-phase vascular recanalization. LR remained the most used model (24/50, 48%), with a C-index of 0.824 (95% CI 0.798-0.850) (Table 4 and Figure 4).

For out-of-hospital mortality, the pooled performance in external validation yielded a C-index of 0.847 (95% CI 0.808-0.887, 95% PI 0.750-0.956), with an SE of 0.71 (95% CI 0.55-0.82) and an SP of 0.76 (95% CI 0.74-0.78). The overall pooled analysis yielded a C-index of 0.812 (95% CI 0.798-0.826, 95% PI 0.693-0.952), with an SE of 0.68 (95% CI 0.63-0.72) and an SP of 0.82 (95% CI 0.78-0.85). Studies on AIS had a pooled C-index of 0.796 (95% CI 0.778-0.813, 95% PI 0.677-0.935), with SE and SP of 0.65 (95% CI 0.59-0.71) and 0.81 (95% CI 0.75-0.85). Studies based on public databases had a pooled C-index of 0.789 (95% CI 0.763-0.815, 95% PI 0.661-0.941), with SE and SP of 0.69 (95% CI 0.62-0.76) and 0.77 (95% CI 0.74-0.80). Studies based on hospital databases had a pooled C-index of 0.824 (95% CI 0.808-0.841, 95% PI 0.712-0.955), with SE and SP of 0.68 (95% CI 0.62-0.74) and 0.84 (95% CI 0.79-0.88). Studies reporting acute-phase vascular recanalization had a pooled C-index of 0.817 (95% CI 0.760-0.879, 95% PI 0.681-0.981), with SE and SP of 0.77 (95% CI 0.69-0.84) and 0.79 (95% CI 0.72-0.85). Internal validation subgroup yielded a C-index of 0.808 (95% CI 0.793-0.823, 95% PI 0.687-0.950), with SE and SP of 0.67 (95% CI 0.62-0.72) and 0.82 (95% CI 0.78-0.85), respectively. The subgroup results were all consistent with the overall trend. In the validation process for out-of-hospital mortality prediction models, those constructed with data sourced from private hospitals show superior overall performance when contrasted with models that rely on data from public datasets.

**Figure 5:**
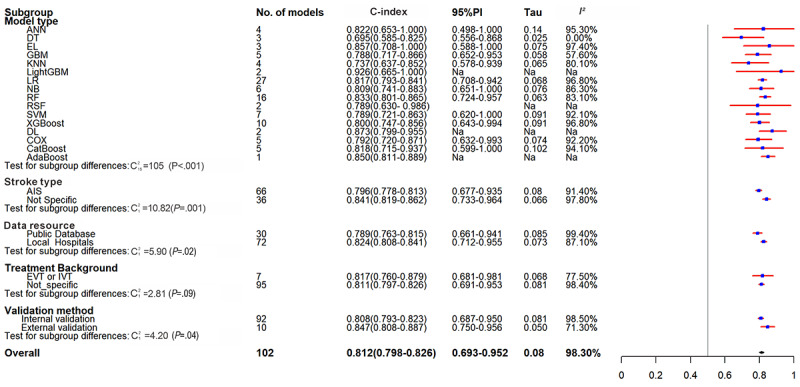
Forest plot for meta-analysis of C-index of predictive models for out-of-hospital mortality in stroke in the validation set. AdaBoost: adaptive boosting; AIS: acute ischemic stroke; ANN: artificial neural network;CatBoost: categorical boosting; COX: Cox proportional hazards; DL: deep learning; DT: decision tree; EL: ensemble learning; EVT: endovascular thrombectomy; GBM: gradient boosting machine; IVT: intravenous thrombolysis; KNN: k-nearest neighbor; LightGBM: light gradient boosting machine; LR: logistic regression; NA:Not applicable; NB: Naive Bayes; PI: prediction interval; RF: random forest; RSF: random survival forest; SVM: support vector machine; XGBoost: extreme gradient boosting.

LR also demonstrated better performance, with a C-index, SE, and SP of 0.817 (95% CI 0.793-0.841, 95% PI 0.708-0.942), 0.74 ( 95% CI 0.69-0.79), and 0.79 (95% CI 0.73-0.84), respectively. DL models for out-of-hospital mortality were created in 2 studies, and they exhibited good discriminatory power (C-index: 0.873, 95% CI 0.799-0.955) but unsatisfactory diagnostic performance (SE 0.48, SP 0.76). (Tables 3 and 4, Figure 5, and Figures S7 and S8 in Multimedia Appendix 2).

**Table 4:** Results of meta-analysis of sensitivity and specificity of machine learning for predicting out-of-hospital mortality in stroke.

Subgroup analysis		Training set					Validation set			
		n	Sample size	SE^a^ (95% CI)	SP^b^ (95% CI)	n		Sample size	SE (95% CI)	SP (95% CI)
**Model**										
	ANN^c^	3	4330	0.58 (0.55-0.61)	0.80 (0.73-0.87)	5		2860	0.61 (0.38-0.80)	0.85 (0.74-0.92)
	DT^d^	—^e^	—	—		4		2217	0.62 (0.24-0.89)	0.72 (0.45-0.89)
	EL^f^	—	—	—	—	3		17,053	0.76 (0.71-0.81)	0.80 (0.78-0.81)
	GBM^g^	2	2859	0.55 (0.39-0.71)	0.84 (0.75-0.93)	5		2076	0.56 (0.38-0.72)	0.80 (0.52-0.94)
	KNN^h^	2	2859	0.45 (0.45-0.46)	0.92 (0.92-0.93)	4		5205	0.53 (0.33-0.72)	0.80 (0.69-0.88)
	AdaBoost^ii^	—	—	—	—	1		1968	0.80	0.81
	LightGBM^j^	1	1388	0.53	0.83	2		2116	0.77 (0.67-0.86)	0.90 (0.82-0.97)
	LR^k^	14	44,677	0.76 (0.69-0.81)	0.80 (0.76-0.83)	21		29,892	0.74 (0.69-0.79)	0.79 (0.73-0.84)
	NB^l^	2	2859	0.51 (0.50-0.52)	0.87 (0.82-0.92)	6		6687	0.68 (0.55-0.79)	0.81 (0.67-0.89)
	RF^m^	3	3313	0.68 (0.52-0.83)	0.81 (0.68-0.93)	11		13,978	0.64 (0.46-0.79)	0.88 (0.72-0.95)
	RSF^n^	—	—	—	—	2		383	0.45 (0.21-0.69)	0.59 (0.42-0.76)
	SVM^o^	2	2859	0.49 (0.41-0.57)	0.81 (0.75-0.86)	7		7225	0.67 (0.53-0.78)	0.87 (0.73-0.94)
	XGBoost^p^	1	1471	0.33	0.98	8		9179	0.70 (0.48-0.86)	0.84 (0.70-0.91)
	DL^q^	2	3821	0.92 (0.83-1.00)	0.99 (0.99-1)	2		1161	0.47 (0.47-0.48)	0.76
	COX^r^	1	425	0.62	0.87	3		1101	0.70 (0.61-0.78)	0.74 (0.70-0.78)
	CatBoost^s^	1	1471	0.68	0.90	3		2741	0.71 (0.52-0.89)	0.78 (0.77-0.79)
**Stroke type**										
	AIS^t^	29	62,174	0.64 (0.56-0.70)	0.89 (0.84-0.93)	49		33,411	0.65 (0.59-0.71)	0.81 (0.75-0.85)
	Not specific	5	10,158	0.78 (0.74-0.80)	0.75 (0.74-0.76)	38		72,431	0.70 (0.64-0.76)	0.83 (0.78-0.87)
**Data resource**										
	Public database	17	55,453	0.64 (0.57-0.71)	0.89 (0.84-0.93)	30		54,088	0.69 (0.62-0.76)	0.77 (0.74-0.80)
	Local hospital	17	16,879	0.69 (0.58-0.78)	0.87 (0.77-0.93)	57		51,754	0.68 (0.62-0.74)	0.84 (0.79-0.88)
**Treatment background**										
	EVT^u^/IVT^v^	—	—	—	—	5		1044	0.77 (0.69-0.84)	0.79 (0.72-0.85)
	Not specific	—	—	—	—	82		104,798	0.67 (0.62-0.72)	0.82 (0.78-0.85)
**Validation method**										
	Internal validation	—	—	—	—	82		103,116	0.67 (0.62-0.72)	0.82 (0.78-0.85)
	External validation	—	—	—	—	5		2726	0.71 (0.55-0.82)	0.76 (0.74-0.78)
Overall		34	72,332	0.66 (0.60-0.72)	0.88 (0.83-0.91)	87		105,842	0.68 (0.63- 0.72)	0.82 (0.78-0.85)

^a^SE: sensitivity.

^b^SP: specificity.

^c^ANN: artificial neural network.

^d^DT: decision tree

^e^Not available.

^f^EL: ensemble learning.

^g^GBM: gradient boosting machine.

^h^KNN: k-nearest neighbor.

^i^AdaBoost: adaptive boosting.

^j^LightGBM: light gradient boosting machine.

^k^LR: logistic regression.

^l^NB: Naive Bayes.

^m^RF: random forest.

^n^RSF: random survival forest.

^o^SVM: support vector machine.

^p^XGBoost: extreme gradient boosting.

^q^DL: deep learning.

^r^COX: Cox proportional hazards.

^s^CatBoost: categorical boosting.

^t^AIS: acute ischemic stroke.

^u^EVT: endovascular thrombectomy.

^v^IVT: intravenous thrombolysis.

**Figure 6:**
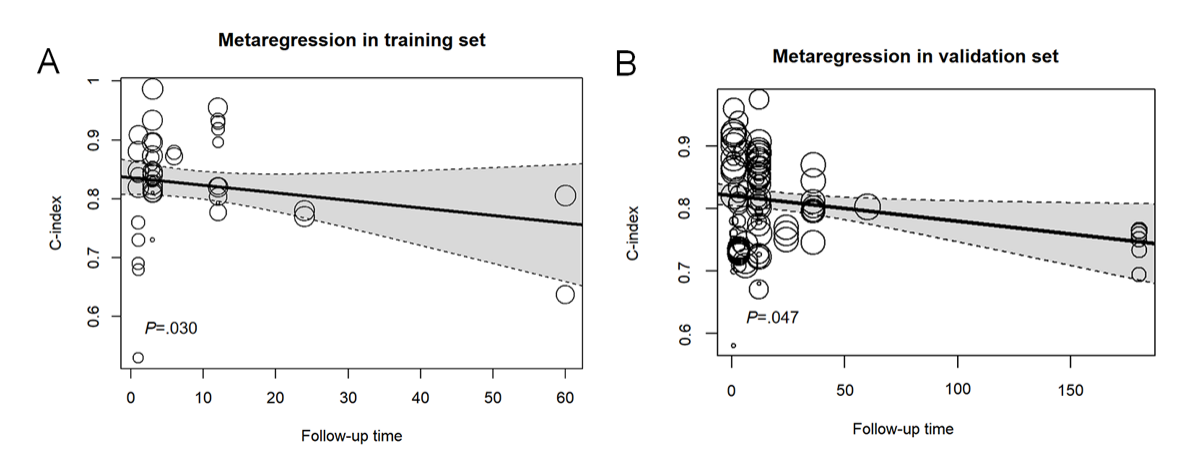
Metaregression bubble plot of follow-up time in the predictive models for (A) training set and (B) validation set.

Surprisingly, only a few studies adopted survival analysis modeling (only 1 COX [Cox Proportional Hazards] model in the training set, and only 5 COX models and 2 random survival forest models in the validation set). Furthermore, metaregression revealed a gradual decline in the predictive performance of the LR models and a sustained performance of the RF and XGBoost models (Figure 6A and B and Figures S9-S11 in Multimedia Appendix 2).

### Modeling Variables

**Figure 7:**
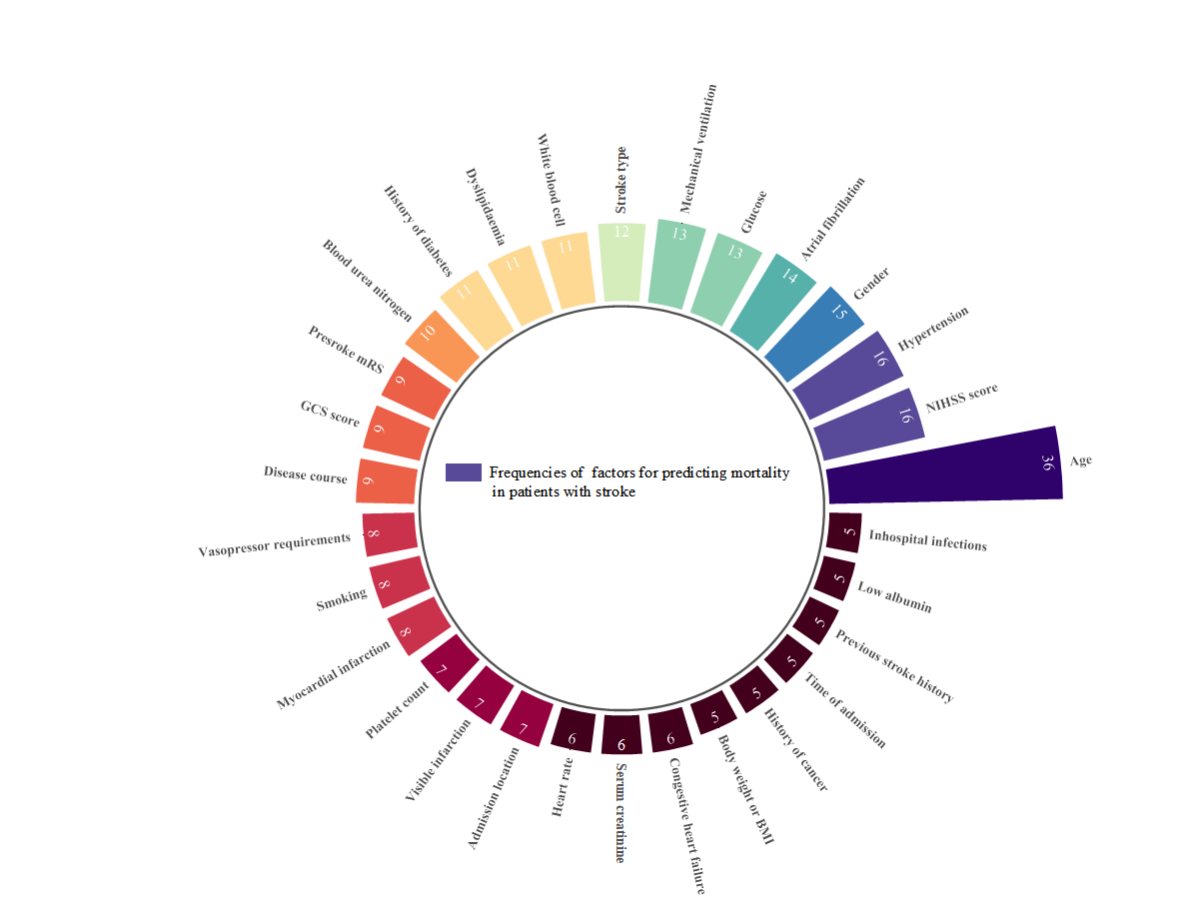
Machine learning models established by predictors for poststroke mortality. GCS: Glasgow Coma Scale; mRS: modified Rankin Scale; NIHSS: National Institutes of Health Stroke Scale.

Of the 155 clinical variables included, high-frequency variables (>5 occurrences) are shown in Figure 7, with age (n=36), National Institutes of Health Stroke Scale (NIHSS) score (n=16), and hypertension (n=16) as the top 3. These variables were distributed across comorbidities (smoking, previous stroke history, cancer, and in-hospital infections), acute severity of illness (NIHSS, Glasgow Coma Scale, and premorbid modified Rankin Scale [mRS] scores), and admission characteristics (time of admission). Few studies involved biomarkers such as platelet-to-neutrophil ratio (PNR) (n=1) and neutrophil-percentage-to-albumin ratio (NPAR) (n=1).

### Small-Study Effect Assessment

As revealed by the funnel plot asymmetry (with fewer studies skewing toward lower performance values) and significant results of Egger tests (*P*<.001), significant small study effects were present in the included studies on out-of-hospital mortality (Figure S13 in Multimedia Appendix 2). Meanwhile, no small study effects were present in the included studies on in-hospital mortality and LR and RF models (Figures S12-S15 in Multimedia Appendix 2).

### SE Analysis

SE analyses were performed after controlling for cohort overlap, yielding pooled C-indexes based on selected studies: For in-hospital mortality, Ouyang et al [50] (MIMIC-III), Cummins et al [48] (MIMIC-IV), Huang et al [58] (eICU-CRD), and Wang et al [43] (SSNAP) were selected; for out-of-hospital mortality, Li et al [81] (MIMIC-III) and Zhu et al [96] (MIMIC-IV) were selected as representatives. Thus, the pooled C-indexes for in-hospital and out-of-hospital mortality prediction models were 0.801 (95% CI 0.777-0.828, 95% PI 0.682-0.992) and 0.825 (95% CI 0.811-0.840, 95% PI 0.711-0.957), respectively. Similar to the primary analysis, these results indicated that the main findings of this meta-analysis were robust to cohort overlap. From further single original studies, 16 and 32 optimal models for predicting in-hospital and out-of-hospital mortality were retained, respectively, with pooled C-indexes of 0.852 (95% CI 0.811-0.895, 95% PI 0.699-1.000) and 0.842 (95% CI 0.817-0.868, 95% PI 0.720-0.986), respectively (Figures S16 [27,43,45,48,50,51,58,67,68,70,76,83,99,101-103] and S17 [29, 40, 42, 44, 46, 49, 53-56, 59, 60, 65, 67, 69, 71, 72, 74, 75, 77-82, 86, 89, 91, 93, 95, 96, 100] in Multimedia Appendix 2). Besides, 5 and 6 external validation studies were included for in-hospital and out-of-hospital mortality, respectively. They yielded pooled C-indexes of 0.785 (95% CI 0.698-0.884, 95% PI 0.594-1.000) and 0.853 (95% CI 0.781-0.931, 95% PI 0.708-1.000), respectively (Figures S18 [50,51,53,63,99] and S19 [29,54,56,71,74,79] in Multimedia Appendix 2).

## Discussion

### Main Findings

To our knowledge, this is the first meta-analysis to evaluate ML as a promising tool for predicting stroke mortality. Different from previous reviews, we conducted subgroup analyses across multiple dimensions, including the model type, patient source, and time. Sixty-eight studies were included in this study, involving numerous ML models for predicting poststroke mortality. Despite methodological concerns of these studies, the most rigorous evidence on generalizability derived from the externally validated subset. The results demonstrated favorable discriminatory power, with pooled C-indices of approximately 0.73 for in-hospital and 0.85 for out-of-hospital mortality, alongside maintaining balanced SE and SP for both in-hospital and out-of-hospital mortality.

The predictive performance of LR models for long-term out-of-hospital mortality might gradually decline, while RF and XGBoost achieved a robust and sustained performance. Subgroup analyses suggested that the models for AIS and the presence or absence of acute-phase vascular recanalization had no great difference from the overall model. Accounting for approximately 50% of the total, LR had slightly inferior performance to other ML methods. Besides, the PI for the C-index for in-hospital or out-of-hospital mortality did not cover the reference line, suggesting that the LR model consistently outperforms random prediction across clinical settings and possesses translational potential. Notably, the PIs were broader in some subgroups due to the limited number of included studies, so further validation is needed in the future. To sum up, ML holds significant value in predicting both in-hospital and out-of-hospital stroke mortality. These findings offer an evidence basis for developing precise, dynamic clinical risk prediction tools in the future.

### Comparison With Previous Reviews

Previous reviews have also investigated ML for early judgment of stroke prognosis. Yang et al [104] conducted a meta-analysis of AI models for predicting ischemic stroke outcomes by binary prediction of 90-day mRS, with 7 studies included. They found a pooled area under the curve (AUC) up to 0.872 of the fixed-effects model, confirming the value of AI in predicting a good prognosis of stroke (mRS ≤2). However, they ignored the assessment of mortality (mRS=6). Schwartz et al [105] and Wang et al [106] included 25 and 7 original studies, respectively, and argued that ML achieves better predictive effects on short- and long-term stroke mortality. However, they performed only a qualitative assessment, thus quantitative evidence was lacking. In addition, available studies on ML for predicting stroke mortality do not classify prediction models and consider the influence of time factors on the model accuracy, generating concerns about heterogeneity in population and methodology. Therefore, we expanded the model performance beyond previous systematic reviews by quantitatively synthesizing evidence from external validations to evaluate model generalizability, in addition to assessing the goodness-of-fit in training sets.

### Modeling Variables

Modeling variables (clinical features, radiomics features, genetic information, and microbiomics) also constituted a challenge to this study. Due to the critical conditions of severe stroke, some examinations such as magnetic resonance imaging are difficult and consume vast human and material resources. Only 2 studies used radiomics [28,54], which relied primarily on interpretable clinical features. We synthesized interpretable clinical features and confirmed that age, NIHSS score, and hypertension were the most used modeling variables, consistent with previous reviews [21,105]. Meanwhile, some studies also focused on specific laboratory indicators. NPAR combines inflammation with nutrient metabolic status, which is more reflective of the body’s acute stress response and overall reserve status [107]. Therefore, NPAR possesses greater predictive power. Chunjuan et al [62] created a nomogram using NPAR and the systematic inflammatory response index for predicting stroke mortality, with an AUC of 0.637. Chung et al [108] proposed that NPAR in combination with simple clinical indicators demonstrates excellent predictive performance for severe illness, with an AUC (0.929) comparable with the Sequential Organ Failure Assessment score, which enables better clinical judgment and interventions. Geng et al [93] adopted PNR to reflect thrombotic and inflammatory status, in which AUC increases from 0.736 (PNR alone) to 0.888 (PNR incorporating age and NIHSS scores). Therefore, the association of changes in some variables with the mortality risk can be observed in the future.

For intensive care unit patients, simple biomarkers and clinical parameters can be combined with laboratory tests to predict the disease severity. In addition, radiomics can be further introduced to reflect individual neuropathologic features and predict prognosis [109]. Besides, models from individual hospitals generally yielded higher C-indexes. The possible reason is as follows: electronic health records at medical centers often provide more detailed, abundant, and clinically relevant predictor variables than public databases covering broad populations, enabling models to capture more complex prediction patterns [110,111].

### Model Selection

Besides modeling variables, the model types (well-interpretable LR, COX, competing risk, and DT) should also be taken into account. These models can quantify the association of variables with risk and thus assist in determining the specific scoring tools, but their accuracy is unsatisfactory. In addition, traditional ML displays high accuracy [112], but its interpretability is challenging. Although ML models interpret “black-box models” using various Shapley additive explanations (SHAP) diagrams at the local and global levels in some studies, the SHAP value under its computing paradigm provides the mean contribution of features in the global scope rather than local logical explanations for specific predictions. Due to this essential attribute, SHAP is difficult to meet the requirements for causality and fidelity in high-reliability scenarios such as clinical diagnosis. Consequently, SHAP values often fail to accurately capture the actual impact of features and may generate misleading feature importance rankings, which restricts their credibility and applicability in high-risk decision-making [113,114]. In the subgroup analysis, LR was dominant in the included studies, with favorable SE and C-index, suggesting that routine interpretable models can early predict stroke mortality. Because of its theoretical simplicity and ease of implementation, LR is widely favored in clinical prediction models [115]. In particular, datasets may be relatively simple and exhibit linear trends in the distribution when data predicting stroke mortality are primarily from clinical history and testing. Therefore, the performance of LR is often comparable with that of complex ML models [112,116]. Meanwhile, LR can quantify the association between various influencing factors and mortality risk and further create a nomogram to better visualize results and thus develop convenient online prediction tools [117]. Thus, despite growing attention to ML, LR remains dominant in data analysis.

Besides predictive accuracy, model interpretability must also be considered in current clinical applications. Generally, highly interpretable models, such as LR, COX, and DT, exhibit limitations in predictive accuracy. In contrast, less interpretable models, such as RF, support vector machine, neural networks, and DL, possess superior accuracy but low interpretability due to their “black-box” property [118,119]. Consequently, less interpretable models face challenges in clinical practice, including limited use by health care providers and difficulty in patient management [120]. Therefore, highly interpretable models should be prioritized. If their accuracy fails to meet clinical requirements, model interpretation techniques (eg, Grad-CAM and LIME) should be introduced to enhance understanding and trust in complex model outputs while maintaining predictive performance [121]. Such interpretable methods are crucial for advancing AI integration and application in clinical settings [122,123]. Besides, DL has high efficiency for image processing and excellent time effect capture ability when processing time series data [124,125], so it has the potential for judging stroke site and time. However, DL is less applied to the early prediction of poststroke mortality, possibly attributed to the difficulty in its interpretability and high requirements for techniques [126]. In the future, DL using multimodal data may be more accurate in prognostic prediction of stroke.

Our meta-analysis revealed a gradual decline in the predictive performance of the overall model and LR model for long-term mortality, consistent with previous reviews [127,128]. The reason may be related to variations in clinical background, alterations in body status, and introduction of time-varying confounders. Notably, RF achieved a robust performance in long-term mortality prediction, with high accuracy, suggesting that RF may be an effective ML model for predicting stroke mortality. Given rehabilitation progresses postdischarge, condition and complications of patients with stroke often present dynamic evolution, so the reliability of AI and ML models relying solely on information at a single time point during hospitalization is restricted in prediction and assessment. Therefore, real-time collection and analysis of patient data are crucial for accurately identifying individual phenotypic changes [129]. Establishing a collaborative real-time data network has become a key foundation for long-term, dynamic clinical decision-making [19]. Moreover, for predicting out-of-hospital mortality, survival analysis modeling was adopted in very few studies. The clinical significance greatly varied between survival analysis modeling and nonsurvival analysis modeling, while the 2 had a minor difference in short-term follow-up; dynamic mortality risk prediction was often a focus in clinical practice, which could be better achieved by survival analysis modeling, with a statistical basis for decision-making [130]. In contrast, nonsurvival analysis modeling was difficult to achieve dynamic mortality risk prediction. Although LR dominates in the analysis currently, dynamic prediction models should be more considered for long-term mortality in the future to develop predictive strategies in real time.

### Prospects and Outlook

The potential of ML for predicting stroke mortality was confirmed in this study, but several technical challenges remain. The population is unbalanced data and the dataset balance is a serious challenge [131]. This meta-analysis found a mortality of about 13% from public databases and 11% from hospital databases. However, the impact of data imbalance was considered in few studies. We synthesized SE and SP of models of unbalanced data and found that despite good performance in predicting negative events (ie, survival), these models were less accurate in predicting positive events (ie, mortality) and were difficult to produce clinical benefits [132]. Therefore, the impact of data imbalance should be taken into account in future modeling.

Another challenge arises from the limited availability and divergent performance patterns of external validation evidence. For in-hospital mortality, the performance in external validation was more conservative than the overall performance and performance in internal validation, probably due to optimistic bias in high-dimensional prognostic settings and simple internal validations [133,134], However, the external validation set remained clinically meaningful and represented an appropriate basis for assessing model generalizability. Conversely, regarding out-of-hospital mortality, the external validation set had more satisfactory C-index than internal validation. This phenomenon was observed in all the 4 included studies [29,54,71,79], which might be related to the fact that patients had more severe conditions, higher mortality, and greater case-mix variation in external validation, thus enhancing the model’s discriminatory power [29,135]. However, due to the limited number and potential representativeness concerns [136], the generalizability of this finding requires cautious interpretation.

This meta-analysis revealed significant small study effects in out-of-hospital mortality, with small sample studies tending to report lower C-index values. This may also be attributed to limited standardized data, introducing data noise that attenuated the model’s apparent performance [137]. Furthermore, the optimal performance still generally fell below the average of large sample studies. Small sample studies also possessed a narrower case-mix (ie, more homogeneous population), and established evidence suggests that the C-index is naturally lower in homogeneous populations [39]. The lower pooled C-index observed in small sample studies likely reflected clinical heterogeneity or implementation quality, rather than publication bias or outcome reporting bias [138]. Consequently, the inclusion of these studies probably yielded a conservative underestimate of the true performance of robust models developed with sufficient resources. Also, our findings of subgroup analyses should be interpreted with caution. Since algorithm types were not randomly assigned across studies, these differences were observed and might be confounded by other study-level factors rather than the algorithms themselves. Therefore, these results represented associations rather than definitive causal evidence [139].

Furthermore, the translation of these models into clinical practice requires rigorous validation. The vast majority of included studies did not distinguish independent test sets and lacked valid external validation. As a result, the system suitability of the AI-based clinical decision support system was limited in clinical workflows [21]. Therefore, future research should conduct external validation of algorithms by program standardization in larger prospective cohorts and compare multiple existing models by external validation to determine the most useful one [140]. Moreover, the models can be compared with relevant clinicians’ judgment, such as providing clinicians (including intensivists and neurologists) with routine clinical information. Additional metrics can also be assessed, such as family acceptance of AI results, resource utilization and planning, and adjustments to subsequent medical plans [141]. Additionally, these algorithms still need improvement to identify mortality in all at-risk populations (low-, medium-, and high-risk populations), and they should be trained by a large number of outlier samples or oversampling techniques, such as random or boundary oversampling or adaptive synthetic sampling. These approaches will facilitate the critical deployment of AI in stroke mortality prediction.

### Advantages and Limitations

#### Advantages

This meta-analysis first quantitatively reviewed the value of ML in predicting in-hospital and out-of-hospital mortality in stroke. The findings are expected to offer an accurate framework for poststroke mortality risk assessment and clinical decision-making.

#### Limitations

Some limitations are worth noting. First, this meta-analysis exhibited great heterogeneity, which can be attributed to the variations in algorithms and study populations. Few studies reporting acute-phase vascular recanalization were included, and most of the studies did not classify the treatment background in detail, which may affect the overall performance assessment. Also, data mostly came from available cohort studies or conventional registries, and some were single-center studies, introducing some bias and restricting the accuracy. Therefore, prospective multicenter studies with detailed subgroup analyses (including treatment backgrounds) are needed. Second, outcome metrics were missing in a small number of studies, so reasonable estimation was implemented, but the model’s predictive accuracy estimates were still affected. Third, internal validation was used in the majority of the studies and external validation in only 20% of the studies, weakening the model’s extrapolation capability. Fourth, multiple studies included were from a small number of large public databases, potentially leading to cohort overlap. Although the limited impact of potential cohort overlap on the pooled effect was confirmed by the SE analysis, we recognized that this method could not fully eliminate residual overfitting bias, given that such bias may arise from systematic similarities in patient populations and clinical protocols across these databases [142]. This concealed overlap could still inflate the summary effect estimates and complicate the interpretation of heterogeneity. Therefore, the extrapolation of our conclusions to more heterogeneous clinical settings warrants caution.

### Conclusions

ML demonstrates promising performance in stroke mortality prediction, and some ML models are more valuable in predicting long-term mortality. However, more efforts are needed before application in clinical practice. For example, we should focus on population SP, search for more specific biomarkers, introduce more multimodal data, optimize model balance, and increase external validations. Furthermore, ML models should be used to develop prediction tools for stroke mortality risk applicable in clinical settings to advance their translation into clinical practice. In this way, data-based doctor-patient shared decision-making can be facilitated, and more efficient resource allocation can be achieved in health care institutions, ultimately ameliorating patient outcomes.

## Appendix

Multimedia Appendix 1 PRISMA (Preferred Reporting Items for Systematic Reviews and Meta-Analyses) checklist.

Multimedia Appendix 2 Details of the search strategy.

Multimedia Appendix 3 Results of Prediction model Risk of Bias Assessment Tool assessment of risk of bias in included studies.

## Abbreviations

AI: artificial intelligence
AIS: acute ischemic stroke
AUC: area under the curve
DL: deep learning
eICU-CRD: eICU Collaborative Research Database
HKSJ: Hartung-Knapp-Sidik-Jonkman
LR: logistic regression
MIMIC: Medical Information Mart for Intensive Care
ML: machine learning
mRS: modified Rankin Scale
NIHSS: National Institutes of Health Stroke Scale
NPAR: neutrophil-percentage-to-albumin ratio
PI: prediction interval
PNR: platelet-to-neutrophil ratio
PRISMA: Preferred Reporting Items for Systematic Reviews and Meta-Analyses
PRISMA-S: Preferred Reporting Items for Systematic Reviews and Meta-Analyses literature search extension
PROBAST: Prediction model Risk of Bias Assessment Tool
RF: random forest
RoB: risk of bias
SE: sensitivity
SHAP: Shapley additive explanations
SP: specificity
SSNAP: Sentinel Stroke National Audit Programme
XGBoost: extreme gradient boosting

## Data Availability

All data generated or analyzed during this study are included in this published paper and its supplementary information files.
