# Supplementary material for: Predictive Value of Machine Learning for Poststroke Mortality Risk: Systematic Review and Meta-Analysis
Source: J Med Internet Res. 2026 Apr 2;28:e83821. doi: 10.2196/83821 (PMC13087561; doi:10.2196/83821)
Supplement: Multimedia Appendix 2 [file jmir_v28i1e83821_app2.docx]

**Multimedia Appendix 2**

**Supplementary Material 1** Literature search strategy

**1.PubMed**

| Search  number | Query | Results |
| --- | --- | --- |
| #1 | ((((((((((((((((Stroke[MeSH Terms]) OR (Stroke[Title/Abstract])) OR (Strokes[Title/Abstract])) OR (Cerebrovascular Accident[Title/Abstract])) OR (Cerebrovascular Accidents[Title/Abstract])) OR (Cerebrovascular Apoplexy[Title/Abstract])) OR (Brain Vascular Accident[Title/Abstract])) OR  (Brain Vascular Accidents[Title/Abstract])) OR (Apoplexy[Title/Abstract])) OR (Brain Infarctions[Title/Abstract])) OR (Brain Infarct[Title/Abstract])) OR (Brain  Infarcts[Title/Abstract])) OR (Brain Infarction[Title/Abstract])) OR (Brain Venous  Infarction[Title/Abstract])) OR (Brain Venous Infarctions[Title/Abstract])) OR (Venous Brain  Infarction[Title/Abstract])) OR (Venous Brain Infarctions[Title/Abstract]) | 413,987 |
| #2 | ((((((((((((((((((((((((((((((((((((((((machine learning[MeSH Terms]) OR (machine  learning[Title/Abstract])) OR (artificial intelligence[Title/Abstract])) OR (Transfer  Learning[Title/Abstract])) OR (prediction model[Title/Abstract])) OR (Deep  learning[Title/Abstract])) OR (deep networks[Title/Abstract])) OR (deep network[Title/Abstract])) OR (CNN[Title/Abstract])) OR (RNN[Title/Abstract])) OR (LSTM[Title/Abstract])) OR  (Generative Adversarial Network[Title/Abstract])) OR (YOLO[Title/Abstract])) OR  (MobileNet[Title/Abstract])) OR (ResNet[Title/Abstract])) OR (AlexNet[Title/Abstract])) OR (VGGNet[Title/Abstract])) OR (GoogLeNet[Title/Abstract])) OR (Ensemble  Learning[Title/Abstract])) OR (risk model[Title/Abstract])) OR (risk score[Title/Abstract])) OR (random forest[Title/Abstract])) OR (neural network[Title/Abstract])) OR (neural  networks[Title/Abstract])) OR (K-Nearest Neighbor[Title/Abstract])) OR (Support vector machine[Title/Abstract])) OR (SVM[Title/Abstract])) OR (Gradient Boosting  Machine[Title/Abstract])) OR (Nomogram[Title/Abstract])) OR (XGBoost[Title/Abstract])) OR (Adaboost[Title/Abstract])) OR (LightGBM[Title/Abstract])) OR (CatBoost[Title/Abstract])) OR  (Gradient Boosting[Title/Abstract])) OR (Decision tree[Title/Abstract])) OR (Regression Trees[Title/Abstract])) OR (Naive Bayesian[Title/Abstract])) OR (Multilayer  perceptron[Title/Abstract])) OR (Bayesian network[Title/Abstract])) OR (Radiomics[Title/Abstract])) OR (Radiomic[Title/Abstract]) | 490,235 |
| #3 | ((((((((((Mortality[MeSH Terms]) OR (Mortality[Title/Abstract])) OR (Mortalities[Title/Abstract])) OR (Case Fatality Rate[Title/Abstract])) OR (Case Fatality Rates[Title/Abstract])) OR (Death  Rate[Title/Abstract])) OR (Death Rates[Title/Abstract])) OR (Survival[Title/Abstract])) OR (Death[Title/Abstract])) OR (OS[Title/Abstract])) OR (CSS[Title/Abstract]) | 3, 118,507 |
| #4 | #1 AND #2 AND #3 | 2,791 |

**2.Cochrane**

| Search  number | Query | Results |
| --- | --- | --- |
| #1 | MeSH descriptor: [Stroke] explode all trees | 18100 |
| #2 | (Stroke):ti,ab,kw OR (Strokes):ti,ab,kw OR (Cerebrovascular Accident):ti,ab,kw OR  (Cerebrovascular Accidents):ti,ab,kw OR (Cerebrovascular Apoplexy):ti,ab,kw OR (Brain  Vascular Accident):ti,ab,kw OR (Brain Vascular Accidents):ti,ab,kw OR (Apoplexy):ti,ab,kw OR  (Brain Infarctions):ti,ab,kw OR (Brain Infarct):ti,ab,kw OR (Brain Infarcts):ti,ab,kw OR (Brain  Infarction):ti,ab,kw OR (Brain Venous Infarction):ti,ab,kw OR (Brain Venous Infarctions):ti,ab,kw  OR (Venous Brain Infarction):ti,ab,kw OR (Venous Brain Infarctions):ti,ab,kw | 82767 |
| #3 | MeSH descriptor: [Machine Learning] explode all trees | 1156 |
| #4 | (machine learning):ti,ab,kw OR (artificial intelligence):ti,ab,kw OR (Transfer Learning):ti,ab,kw  OR (prediction model):ti,ab,kw OR (Deep learning):ti,ab,kw OR (deep networks):ti,ab,kw OR  (deep network):ti,ab,kw OR (CNN):ti,ab,kw OR (RNN):ti,ab,kw OR (LSTM):ti,ab,kw OR  (Generative Adversarial Network):ti,ab,kw OR (YOLO):ti,ab,kw OR (MobileNet):ti,ab,kw OR  (ResNet):ti,ab,kw OR (AlexNet):ti,ab,kw OR (VGGNet):ti,ab,kw OR (GoogLeNet):ti,ab,kw OR  (Ensemble Learning):ti,ab,kw OR (risk model):ti,ab,kw OR (risk score):ti,ab,kw OR (random  forest):ti,ab,kw OR (neural network):ti,ab,kw OR (neural networks):ti,ab,kw OR (K-Nearest  Neighbor):ti,ab,kw OR (Support vector machine):ti,ab,kw OR (SVM):ti,ab,kw OR (Gradient  Boosting Machine):ti,ab,kw OR (Nomogram):ti,ab,kw OR (XGBoost):ti,ab,kw OR  (Adaboost):ti,ab,kw OR (LightGBM):ti,ab,kw OR (CatBoost):ti,ab,kw OR (Gradient  Boosting):ti,ab,kw OR (Decision tree):ti,ab,kw OR (Regression Trees):ti,ab,kw OR (Naive  Bayesian):ti,ab,kw OR (Multilayer perceptron):ti,ab,kw OR (Bayesian network):ti,ab,kw OR  (Radiomics):ti,ab,kw OR (Radiomic):ti,ab,kw | 87137 |
| #5 | MeSH descriptor: [Mortality] explode all trees | 18548 |
| #6 | (Mortality):ti,ab,kw OR (Mortalities):ti,ab,kw OR (Case Fatality Rate):ti,ab,kw OR (Case Fatality  Rates):ti,ab,kw OR (Death Rate):ti,ab,kw OR (Death Rates):ti,ab,kw OR (Survival):ti,ab,kw OR  (Death):ti,ab,kw OR (OS):ti,ab,kw OR (CSS):ti,ab,kw | 275858 |
| #7 | #1 or #2 | 83054 |
| #8 | #3 or #4 | 87141 |
| #9 | #5 or #6 | 275874 |
| #10 | #7 and #8 and #9 | 3752 |

**3.Embase**

| Search  number | Query | Results |
| --- | --- | --- |
| #1 | 'cerebrovascular accident'/exp | 511415 |
| #2 | 'stroke'/exp OR stroke OR strokes:ab,ti OR 'cerebrovascular accident':ab,ti OR 'cerebrovascular accidents':ab,ti OR 'cerebrovascular apoplexy':ab,ti OR 'brain vascular accident':ab,ti OR 'brain  vascular accidents':ab,ti OR apoplexy:ab,ti OR 'brain infarctions':ab,ti OR 'brain infarct':ab,ti OR 'brain infarcts':ab,ti OR 'brain infarction':ab,ti OR 'brain venous infarction':ab,ti OR 'brain venous  infarctions':ab,ti OR 'venous brain infarction':ab,ti OR 'venous brain infarctions':ab,ti | 804682 |
| #3 | 'machine learning'/exp | 600135 |
| #4 | 'machine learning'/exp OR 'machine learning' OR (('machine'/exp OR machine) AND  ('learning'/exp OR learning)) OR 'artificial intelligence':ab,ti OR 'transfer learning':ab,ti OR  'prediction model':ab,ti OR 'deep learning':ab,ti OR 'deep networks':ab,ti OR 'deep network':ab,ti  OR cnn:ab,ti OR rnn:ab,ti OR lstm:ab,ti OR 'generative adversarial network':ab,ti OR yolo:ab,ti OR mobilenet:ab,ti OR resnet:ab,ti OR alexnet:ab,ti OR vggnet:ab,ti OR googlenet:ab,ti OR 'ensemble learning':ab,ti OR 'risk model':ab,ti OR 'risk score':ab,ti OR 'random forest':ab,ti OR 'neural  network':ab,ti OR 'neural networks':ab,ti OR 'k-nearest neighbor':ab,ti OR 'support vector  machine':ab,ti OR svm:ab,ti OR 'gradient boosting machine':ab,ti OR nomogram:ab,ti OR  xgboost:ab,ti OR adaboost:ab,ti OR lightgbm:ab,ti OR catboost:ab,ti OR 'gradient boosting':ab,ti OR 'decision tree':ab,ti OR 'regression trees':ab,ti OR 'naive bayesian':ab,ti OR 'multilayer  perceptron':ab,ti OR 'bayesian network':ab,ti OR radiomics:ab,ti OR radiomic:ab,ti | 857641 |
| #5 | 'mortality'/exp | 1597807 |
| #6 | 'mortality'/exp OR mortality OR mortalities:ab,ti OR 'case fatality rate':ab,ti OR 'case fatality  rates':ab,ti OR 'death rate':ab,ti OR 'death rates':ab,ti OR survival:ab,ti OR death:ab,ti OR os:ab,ti OR css:ab,ti | 4786156 |
| #7 | #2 OR #3 | 804682 |
| #8 | #4 OR #5 | 857641 |
| #9 | #6 OR #7 | 4884532 |
| #10 | #7 AND #8 AND #9 | 6869 |

**4.Web of Science**

| Search  number | Query | Results |
| --- | --- | --- |
| #1 | Stroke (Topic) OR Strokes (Topic) OR "Cerebrovascular Accident" (Topic) OR  "Cerebrovascular Accidents" (Topic) OR "Cerebrovascular Apoplexy" (Topic) OR "Brain  Vascular Accident" (Topic) OR "Brain Vascular Accidents" (Topic) OR Apoplexy (Topic) OR  "Brain Infarctions" (Topic) OR "Brain Infarct" (Topic) OR "Brain Infarcts" (Topic) OR "Brain  Infarction" (Topic) OR "Brain Venous Infarction" (Topic) OR "Brain Venous Infarctions"  (Topic) OR "Venous Brain Infarction" (Topic) OR "Venous Brain Infarctions" (Topic) and Preprint Citation Index (Exclude - Database) | 1069795 |
| #2 | "machine learning" (Topic) OR "artificial intelligence" (Topic) OR "Transfer Learning" (Topic) OR "prediction model" (Topic) OR "Deep learning" (Topic) OR "deep networks" (Topic) OR  "deep network" (Topic) OR CNN (Topic) OR RNN (Topic) OR LSTM (Topic) OR "Generative Adversarial Network" (Topic) OR YOLO (Topic) OR MobileNet (Topic) OR ResNet (Topic) OR AlexNet (Topic) OR VGGNet (Topic) OR GoogLeNet (Topic) OR "Ensemble Learning" (Topic) OR "risk model" (Topic) OR "risk score" (Topic) OR "random forest" (Topic) OR "neural  network" (Topic) OR "neural networks" (Topic) OR "Support vector machine" (Topic) OR SVM | 4083889 |
|  | (Topic) OR "Gradient Boosting Machine" (Topic) OR Nomogram (Topic) OR XGBoost (Topic) OR Adaboost (Topic) OR LightGBM (Topic) OR CatBoost (Topic) OR "Gradient Boosting"  (Topic) OR "Decision tree" (Topic) OR "Regression Trees" (Topic) OR "Naive Bayesian"  (Topic) OR "Multilayer perceptron" (Topic) OR "Bayesian network" (Topic) OR Radiomics (Topic) OR Radiomic (Topic) and Preprint Citation Index (Exclude - Database) |  |
| #3 | Mortality (Topic) OR Mortalities (Topic) OR "Case Fatality Rate" (Topic) OR "Case Fatality  Rates" (Topic) OR "Death Rate" (Topic) OR "Death Rates" (Topic) OR Survival (Topic) OR Death (Topic) OR OS (Topic) OR CSS (Topic) and Preprint Citation Index (Exclude - Database) | 6902567 |
| #4 | #1 AND #2 AND #3 and Preprint Citation Index (Exclude - Database) | 7765 |

We imposed no language or other restrictions on any of the searches

**Table S1.** Basic study characteristics.

| No. | Title | PMID | DOI | Author | Publication Year | Country | Study Type | Source of Patients | Type of Stroke | Treatment background | Follow-up Duration | Number of death cases | Total numbers | Modeling variables | Validation cohort generation | Outcomes(Model Type) |
| --- | --- | --- | --- | --- | --- | --- | --- | --- | --- | --- | --- | --- | --- | --- | --- | --- |
| 1 | Machine Learning Predictive Models for Survival in Patients with Brain Stroke | 40453683 | 10.34172/hpp.025.43635 | Solmaz Norouzi | 2025 | Iran | Cohort study(Retrospective) | Single-center study | Not specific |  | 15 year | 227 | 332 | Clinical features | Random division--8：2 | support vector machine (SVM)/COX/XGBoost/DT/GBM/RSF |
| 2 | Machine learning-based scoring model for predicting mortality in ICU-admitted ischemic stroke patients with moderate to severe consciousness disorders | 40170899 | 10.3389/fneur.2025.1534961 | Zhou Zhou | 2025 | China | Cohort study(Retrospective) | Medical Information Mart for Intensive Care IV (MIMIC-IV) | Acute ischemic stroke |  | 90d | 252 | 648 | Clinical features | Random division--7：3 | AutoScore framework |
| 3 | Personalized prediction of mortality in patients with acute ischemic stroke using explainable artificial intelligence | 38902792 | 10.1186/s40001-024-01940-2 | Lingyu Xu | 2024 | China | Cohort study(Retrospective) | Single-center study | Acute ischemic stroke |  | 23d | 79 | 1633 | Clinical features | Random division--8.5：1.5 | (1) light gradient boosting machine (LightGBM), (2) GBM, (3) random forest (RF), (4) K-nearest neighbors (KNN), (5) multi-layer perceptron (MLP), (6) naive Bayes (NB), (7) , and (8) logistic regression (LR) |
| 4 | Risk prediction of 30-day mortality after stroke using machine learning: a nationwide registry-based cohort study | 35624434 | [10.1186/s12883-022-02722-1](https://doi.org/10.1186/s12883-022-02722-1) | Wenjuan Wang | 2022 | China | Cohort study(Retrospective) | SSNAP | Not specific |  | In-Hospital Mortality | 60362 | 488947 | Clinical features | Random division--8：2 | LR, XGBoost |
| 5 | The predictors of death within 1 year in acute ischemic stroke patients based on machine learning | 36908612 | 10.3389/fneur.2023.1092534 | Kai Wang | 2023 | China | Cohort study(Retrospective) | Single-center study | Acute ischemic stroke |  | 365d | 32 | 677 | Clinical features | Random division--7：3 | LR;GBM;XGB;RF;DT;NB |
| 6 | Prediction of mortality in stroke patients using multilayer  perceptron neural networks |  | doi:10.3906/sag-1105-20 | NECDET SÜT | 2012 | Turkey | Case-control study | Single-center study | Not specific |  | In-Hospital Mortality | 127 | 584 | Clinical features | Random division--7：3 | ANN |
| 7 | Predicting mortality in brain stroke patients using neural networks: outcomes analysis in a longitudinal study | 37898678 | 10.1038/s41598-023-45877-8 | Nasrin Someeh | 2023 | Iran | Cohort study(Retrospective) | Single-center study | Not specific |  | 10 year | 228 | 332 | Clinical features | Random division--7：3 | ANN |
| 8 | Machine learning to predict mortality after rehabilitation among patients with severe stroke | 33208913 | 10.1038/s41598-020-77243-3 | Domenico Scrutinio | 2020 | Italy | Cohort study(Retrospective) | Multicenter study | Not specific |  | 3 year | 189 | 1207 | Clinical features | resampling 200 bootstrap | RF;GBM;LR |
| 9 | Predictors of In-Hospital Mortality after Thrombectomy in Anterior Circulation Large Vessel Occlusion: A Retrospective, Machine Learning Study | 39061668 | 10.3390/diagnostics14141531 | Ivan Petrović | 2024 | Serbia | Case-control study | Single-center study | Acute ischemic stroke | Endovascular Treatment | In-Hospital Mortality | 133 | 602 | Clinical features | Random division--7：3 | LR;RF;GB;XGBoost |
| 10 | In-Hospital Mortality Prediction among Intensive Care Unit Patients with Acute Ischemic Stroke: A Machine Learning Approach | 40099281 | 10.34133/hds.0179 | Jack A Cummins | 2025 | USA | Cohort study(Retrospective) | MIMIC-IV | Acute ischemic stroke |  | In-Hospital Mortality | 480 | 3489 | Clinical features | Random division--8：2 | RF;LR; XGBoost |
| 11 | A Bayesian network model for predicting post-stroke outcomes with available risk factors | 30245663 | 10.3389/fneur.2018.00699 | Eunjeong Park | 2018 | South Korea | Cohort study(Retrospective) | Single-center study | Acute ischemic stroke |  | 1 year | 434 | 3605 | Clinical features |  | NB |
| 12 | Interpretable machine learning models for predicting in-hospital death in patients in the intensive care unit with cerebral infarction | 36827826 | 10.1016/j.cmpb.2023.107431 | Yang Ouyang | 2023 | China | Cohort study(Retrospective) | MIMIC-III，eICU Research Institute Database | Acute ischemic stroke |  | In-Hospital Mortality | 662 | 4338 | Clinical features | Random division--7：3；External validation(MIMIC-III) | KNN/RF/XGBoost/SVM/ANN |
| 13 | Development and validation of an interpretable machine learning model for predicting in-hospital mortality for ischemic stroke patients in ICU | 40073651 | 10.1016/j.ijmedinf.2025.105874 | Luo Xiao | 2025 | China | Cohort study(Retrospective) | MIMIC-I, Single-center， | Acute ischemic stroke |  | In-Hospital Mortality | 359 | 3225 | Clinical features | Random division--7：3；External validation: single center | LR;KNN;NB;DT;SVM;RF;XGBoost, LightGBM;ANN |
| 14 | Factors associated with 90-day mortality in Vietnamese stroke patients: Prospective findings compared with explainable machine learning, multicenter study | 39302916 | 10.1371/journal.pone.0310522 | [Ton Duy Mai](https://pubmed.ncbi.nlm.nih.gov/?term=) | 2024 | Vietnam | Cohort study(Prospective) | Multicenter study | Not specific |  | 90d | 249 | 2209 | Clinical features | Random division--8：2 | XGBoost |
| 15 | Immune-inflammatory, coagulation, adhesion, and imaging biomarkers combined in machine learning models improve the prediction of death 1 year after ischemic stroke | 34120242 | 10.1007/s10238-021-00732-w | Ana Lucia Cruz Fürstenberger Lehmann | 2022 | Brazil | Cohort study(Retrospective) | Single-center study | Acute ischemic stroke |  | 365d | 22 | 103 | Clinical features | Holdout validation：Training (46.67%), Testing (20%), Holdout (33.33%). | ANN,LR |
| 16 | Imaging-based outcome prediction in posterior circulation stroke | 35257203 | <https://doi.org/10.1007/s00415-022-11010-4> | Helge C. Kniep | 2022 | Germany | Cohort study(Retrospective) | Multicenter study | Acute ischemic stroke | Endovascular Treatment | 90d | 45 | 172 | Image features and clinical features | Nested fivefold cross-validation | LR |
| 17 | Deep learning model integrating radiologic and clinical data to predict mortality after ischemic stroke | 38826743 | 10.1016/j.heliyon.2024.e31000 | Changi Kim | 2024 | Korea | Cohort study(Retrospective) | Multicenter study | Acute ischemic stroke |  | 365d | 205 | 2710 | Image features and clinical features | Random sampling: Training set(n=1,109)；Validation set(n=437)；Internal test set(n=654) | DL;XGBoost;RF |
| 18 | Stroke mortality prediction based on ensemble learning and the combination of structured and textual data | 36805232 | 10.1016/j.compbiomed.2022.106176 | Ruixuan Huang | 2023 | China | Cohort study(Retrospective) | Multicenter study | Not specific |  | 180d | 9855 | 76820 | Clinical features | Random sampling:Training (50%), Testing (25%), Holdout (25%)；External validation:MIMIC-III | ensemble learning based on ML and DL models |
| 19 | Clinical decision support systems for 3-month mortality in elderly patients admitted to ICU with ischemic stroke using interpretable machine learning | 39314817 | 10.1177/20552076241280126 | Jian Huang | 2024 | China | Cohort study(Retrospective) | MIMIC-IV, International Stroke Perfusion Imaging Registry | Acute ischemic stroke |  | 90d | 624 | 1826 | Clinical features | Random division--8：2 | CatBoost；RF；SVM；NN；GBM；KNNs；ANN；NB；XGBoost；LR |
| 20 | Twenty-eight-day in-hospital mortality prediction for elderly patients with ischemic stroke in the intensive care unit: Interpretable machine learning models | 36711330 | 10.3389/fpubh.2022.1086339 | Jian Huang | 2022 | China | Cohort study(Retrospective) | electronic intensive care unit (eICU) Collaborative Research Database (eICU-CRD) | Acute ischemic stroke |  | In-Hospital Mortality | 164 | 1236 | Clinical features | Random division--8：2 | NB,XGBoost,LR |
| 21 | Interpretable machine learning for predicting 28-day all-cause in-hospital mortality for hypertensive ischemic or hemorrhagic stroke patients in the ICU: a multi-center retrospective cohort study with internal and external cross-validation | 37614971 | 10.3389/fneur.2023.1185447 | Jian Huang | 2023 | China | Cohort study(Retrospective) | MIMIC-IV，eICU-CRD | Not specific |  | In-Hospital Mortality | 490 | 4274 | Clinical features | Random division--8：2 | GBM；XGBoost；LR；SVM |
| 22 | Development and Internal Validation of Machine Learning Models to Predict Mortality and Disability After Mechanical Thrombectomy for Acute Anterior Circulation Large Vessel Occlusion | 38000670 | 10.1016/j.wneu.2023.11.060 | Haydn Hoffman | 2024 | USA | Cohort study(Retrospective) | Single-center study | Acute ischemic stroke | Endovascular Treatment | 90d | 130 | 357 | Clinical features | Random division--8：2 | LR;RF |
| 23 | Random forest-based prediction of stroke outcome | 33980906 | 10.1038/s41598-021-89434-7 | Carlos Fernandez-Lozano | 2021 | Spain | Cohort study(Retrospective) | Single-center study | Not specific |  | 90d | 980 | 6022 | Clinical features | 10-fold cross-validation | RF |
| 24 | RGX Ensemble Model for Advanced Prediction of Mortality Outcomes in Stroke Patients | 39600589 | 10.34133/bmef.0077 | J. Fang | 2024 | China | Cohort study(Retrospective) | MIMIC | Acute ischemic stroke |  | In-Hospital Mortality | 347 | 1864 | Clinical features | Random division--8：2 | Ensemble learning; RF;XGBoost；LR; DT |
| 25 | Machine learning consensus clustering for inflammatory subtype analysis in stroke and its impact on mortality risk: a study based on NHANES (1999–2018) | 40276469 | 10.3389/fneur.2025.1562247 | Zhang Chunjuan | 2025 | China | Cohort study(Retrospective) | The National Health and Nutrition Examination Survey (NHANES) database | Not specific |  | 79 month | 369 | 918 | Clinical features |  | LR |
| 26 | Exploring the prognostic impact of triglyceride-glucose index in critically ill patients with first-ever stroke: insights from traditional methods and machine learning-based mortality prediction | 39695656 | 10.1186/s12933-024-02538-y | Chen Yang | 2024 | China | Cohort study(Retrospective) | eICU-CRD; Single-center study | Not specific |  | In-Hospital Mortality | 536 | 3173 | Clinical features | Random division--7：3；External validation：single-center | Light GBM, RF, LR, SVM, ANN;NB;KNN |
| 27 | A novel higher performance nomogram based on explainable machine learning for predicting mortality risk in stroke patients within 30 days based on clinical features on the first day ICU admission | 38849903 | 10.1186/s12911-024-02547-7 | Haoran Chen | 2024 | China | Cohort study(Retrospective) | MIMIC-IV | Not specific |  | 30d | 704 | 2982 | Clinical features | Random division--8：2；External validation：MIMIC-III | LR |
| 28 | Machine Learning Models for Predicting Stroke Mortality in Malaysia: An Application and Comparative Analysis | 38222138 | 10.7759/cureus.50426 | Che Muhammad Nur Hidayat Che Nawi | 2023 | Malaysia | Cohort study(Retrospective) | Multicenter study | Not specific |  | 3year | 272 | 950 | Clinical features | Random division：2：1 | COX;SVM;RSF |
| 29 | Machine learning-based prediction of one-year mortality in ischemic stroke patients | 39569400 | 10.1093/oons/kvae011 | Ahmad Abujaber | 2024 | Qatar | Cohort study(Retrospective) | Single-center study | Acute ischemic stroke |  | 365d | 302 | 8183 | Clinical features | Random division--8：2 | XGBoost;RF;SVM;DT;LR |
| 30 | Predicting Short and Long-Term Mortality After Acute Ischemic Stroke Using EHR | 34218182 | 10.1016 / j.jns.2021.117560 | Vida Abedi | 2022 | USA | Cohort study(Retrospective) | EHR system, Geisinger Quality database, and the Social Security Death database | Acute ischemic stroke |  | 2 year | 1,797 | 7144 | Clinical features | Random division--8：2 | LR,XGB,RF |
| 31 | Development of a Predictive Nomogram for Intra-Hospital Mortality in Acute Ischemic Stroke Patients Using LASSO Regression | 39139210 | 10.2147/CIA.S471885 | Li Zhou | 2024 | China | Case-control study | Single-center study | Acute ischemic stroke |  | In-Hospital Mortality | 40 | 422 | Clinical features | Random division--7：3 | LR |
| 32 | Nomogram to Predict Mortality of Endovascular Thrombectomy for Ischemic Stroke Despite Successful Recanalization | 31973604 | 10.1161/JAHA.119.014899 | Xiaohao Zhang | 2020 | China | Cohort study(Retrospective) | Multicenter study | Acute ischemic stroke | Endovascular Treatment | 90d | 120 | 641 | Clinical features | 10‐fold cross‐validation;external validation：multi-center | LR |
| 33 | An mNUTRIC-based nomogram for predicting the in-hospital death risk in patients with acute stroke | 35411025 | 10.1038/s41430-022-01127-0 | Rui-xin Zhang | 2022 | China | Case-control study | Single-center study | Not specific |  | In-Hospital Mortality | 69 | 268 | Clinical features | Bootstrap resampling | LR |
| 34 | Ultrasonic optic nerve sheath diameter as a new predictor for the mortality of patients with large hemispheric infarction | 39748001 | 10.1038/s41598-024-84720-6 | Jie Zhang | 2025 | China | Cohort study(Prospective) | Multicenter study | Acute ischemic stroke |  | 90d | 175 | 419 | Clinical features | Random division--7：3；External validation：multi-center | LR |
| 35 | Predictions for functional outcome and mortality in acute ischaemic stroke following successful endovascular thrombectomy | 38932996 | 10.1136 / bmjno - 2024 - 000707 | Minyan Zeng | 2024 | China | Cohort study(Retrospective) | Single-center study | Acute ischemic stroke | Endovascular Treatment | 3 year | 240 | 978 | Clinical features | Bootstrap resampling | LR |
| 36 | Predicting 10-day mortality in patients with strokes using neural networks and multivariate statistical methods | 24674954 | 10.1016/j.jstrokecerebrovasdis.2013.12.018 | Guner ¸ Celik | 2014 | Turkey | Case-control study | Single-center study | Not specific |  | In-Hospital Mortality | 293 | 968 | Clinical features | 5-fold cross-validation | XGboost;ANN;LR;Discriminant analysis |
| 37 | Nomogram to predict 6-month mortality in acute ischemic stroke patients treated with endovascular treatment | 38249750 | 10.3389/fneur.2023.1330959 | Rui Wen | 2024 | China | Cohort study(Retrospective) | Single-center study | Acute ischemic stroke | Endovascular Treatment | 180d | 124 | 434 | Clinical features | External validation:single center | LR |
| 38 | Predicting 10-year stroke mortality: development and validation of a nomogram | 34406610 | 10.1007/s13760-021-01752-9 | Weronika A Szlachetka | 2022 | UK | Cohort study(Retrospective) | The Norfolk and Norwich Stroke and TIA Register (NNSTR) | Acute ischemic stroke |  | 10 year | 4879 | 10366 | Clinical features | Bootstrap resampling | LR |
| 39 | A nomogram for predicting the in-hospital mortality after large hemispheric infarction | 31884967 | 10.1186/s12883-019-1571-4 | Wenzhe Sun | 2019 | China | Case-control study | Single-center study | Acute ischemic stroke |  | In-Hospital Mortality | 58 | 158 | Clinical features |  | LR |
| 40 | Derivation and validation of a simple risk score for predicting 1-year mortality in stroke | 17934885 | 10.1007/s00415-007-0555-2 | O G Solberg | 2007 | Norway | Cohort study(Retrospective) | Single-center study | Not specific |  | 365d | 274 | 737 | Clinical features | Random division--6：4 | LR |
| 41 | Predictors of death within 6 months of stroke onset: A model with Barthel index, platelet/lymphocyte ratio and serum albumin | 33378600 | 10.1002/nop2.754 | Ling Sha | 2020 | China | Cohort study(Retrospective) | Single-center study | Not specific |  | 180d | 49 | 210 | Clinical features | Bootstrap resampling | LR |
| 42 | Technical Risk Stratification Nomogram Model for 90-Day Mortality Prediction in Patients With Acute Basilar Artery Occlusion Undergoing Endovascular Thrombectomy: A Multicenter Cohort Study | 38471827 | 10.1161/JAHA.123.032107 | Heng Ni | 2024 | China | Cohort study(Retrospective) | Multicenter study | Acute ischemic stroke | Endovascular Treatment | 90d | 68 | 242 | Clinical features | Bootstrap 1000;external validation:multi-center | LR |
| 43 | Early Prediction of One-Year Mortality in Ischemic and  Haemorrhagic Stroke | 32044222 | 10.1016/j.jstrokecerebrovasdis.2020.104667 | J. Liljehult | 2020 | Denmark | Cohort study(Retrospective) | Single-center study | Not specific |  | 365d | 186 | 1031 | Clinical features | bootstrap cross-validation(63.2%) | LR |
| 44 | A novel nomogram to predict mortality in patients with stroke: a survival analysis based on the MIMIC-III clinical database | 35387672 | 10.1186/s12911-022-01836-3 | Xiao-Dan Li | 2022 | China | Cohort study(Retrospective) | MIMIC-III | Not specific |  | 360d | 285 | 767 | Clinical features | Random division--7：3 | LR |
| 45 | Stroke Measures Analysis of pRognostic Testing-Mortality nomogram predicts long-term mortality after ischemic stroke | 39150095 | 10.1177/17474930241278808 | Tae Jung Kim | 2025 | China | Cohort study(Retrospective) | The administrative claims database | Acute ischemic stroke |  | 5 year | 9108 | 42207 | Clinical features | Random division--7：3 | LR |
| 46 | Derivation and validation of in-hospital mortality prediction models in ischaemic stroke patients using administrative data | 23429000 | 10.1159/000346090 | Jason Lee | 2013 | Japan | Case-control study | Multicenter study | Acute ischemic stroke |  | In-Hospital Mortality | 944 | 21445 | Clinical features | Random division--5：5 | LR |
| 47 | Prediction of long-term mortality in patients with ischemic stroke based on clinical characteristics on the first day of ICU admission: An easy-to-use nomogram | 37122313 | 10.3389/fneur.2023.1148185 | Jin Guangyong | 2014 | China | Cohort study(Retrospective) | MIMIC-IV | Acute ischemic stroke |  | 365d | 457 | 2089 | Clinical features | Random division--7：3 | LR |
| 48 | Development and verification of a nomogram for predicting short-term mortality in elderly ischemic stroke populations | 37537270 | 10.1038/s41598-023-39781-4 | G. Jin | 2023 | China | Cohort study(Retrospective) | MIMIC-IV | Acute ischemic stroke |  | In-Hospital Mortality | 332 | 1259 | Clinical features | Random division--7：3 | LR |
| 49 | Predicting Early Mortality of Acute Ischemic Stroke: Score-Based Approach |  | 10.1161/strokeaha.118.022863 | Thomas Gattringer | 2018 | Australia | Cohort study(Retrospective) | The nationwide Austrian Stroke Unit Registry | Acute ischemic stroke |  | In-Hospital Mortality | 1846 | 86794 | Clinical features | Bootstrapping 2000；temporal validation | LASSO |
| 50 | Development and validation of a risk score to predict 30-day mortality in patients with atrial fibrillation-related stroke: GPS-GF score | 29544401 | 10.1080/01616412.2018.1451431 | Hua Gao | 2018 | China | Cohort study(Retrospective) | Multicenter study | Acute ischemic stroke |  | 30d | 200 | 1077 | Clinical features | Random division--2：1 | LR |
| 51 | Development and validation of a nomogram for predicting 28-day mortality in patients with ischemic stroke | 38656987 | 10.1371/journal.pone.0302227 | Lingyan Fang | 2024 | China | Cohort study(Retrospective) | MIMIC-IV | Acute ischemic stroke |  | In-Hospital Mortality |  | 2089 | Clinical features | Random division--7：3 | LR |
| 52 | A nomogram to predict the probability of mortality after first-ever acute manifestations of cerebral small vessel disease | 29406922 | 10.1016/j.jns.2017.12.020 | Cappellari Manuel | 2018 | Italy | Cohort study(Retrospective) | Single-center study | Acute ischemic stroke |  | 90d | 38 | 288 | Clinical features | 1000 samples bootstrap and cross-validation | LR |
| 53 | Derivation and external validation of a case mix model for the standardized reporting of 30-day stroke mortality rates | 25293667 | 10.1161/STROKEAHA.114.006451 | Benjamin D Bray | 2014 | UK | Cohort study(Retrospective) | SSNAP; the South London Stroke Register (SLSR) | Not specific |  | In-Hospital Mortality | 3593 | 27,169 | Clinical features | Random division--1：2；External validation(SLSR) | LR |
| 54 | Incorporating Stroke Severity Into Hospital Measures of 30-Day Mortality After Ischemic Stroke Hospitalization | 28954922 | 10.1161/STROKEAHA.117.017960 | Jennifer Schwartz | 2017 | USA | Cohort study(Retrospective) | Medicare fee-for-service administrative claims and the AHA/ASA GWTG-Stroke registry) | Acute ischemic stroke |  | 30d | 27299 | 188975 | Clinical features | Random division--5：5 | LR |
| 55 | External validation of risk prediction models for post-stroke mortality in Berlin | 40480675 | 10.1136/bmjopen-2024-089320 | Lukas Reitzle | 2025 | Germany | Cohort study(Retrospective) | Multicenter study | Not specific |  | 30d | 763 | 9810 | Clinical features | External validation | Bray |
| 56 | Stacking ensemble learning model to predict 6-month mortality in ischemic stroke patients | 36253488 | 10.1038/s41598-022-22323-9 | Hwangbo Lee | 2022 | South Korea | Cohort study(Retrospective) | International Stroke Trial database | Acute ischemic stroke |  | 180d | 1285 | 8787 | Clinical features | Random division--7：3 | Ensemble learning: XGBoost;KNN; SVM |
| 57 | A Dynamic Model for Predicting Survival up to 1 Year After Ischemic Stroke | 32912566 | 10.1016/j.jstrokecerebrovasdis.2020.105133 | Yan Huang | 2020 | China | Cohort study(Retrospective) | SLSR | Acute ischemic stroke |  | 365d | 1210 | 4315 | Clinical features | Temporal validation | Cox |
| 58 | Association of platelet-to-neutrophil ratios with 1-year outcome and mortality in patients with acute ischemic stroke | 36529389 | 10.1016/j.neulet.2022.137016 | Leiyu Geng | 2025 | China | Cohort study(Prospective) | Single-center study | Acute ischemic stroke |  | 365d | 123 | 718 | Clinical features |  | Cox |
| 59 | Interpretability Analysis of One-Year Mortality Prediction for Stroke Patients Based on Deep Neural Network | 34714758 | 10.1109/JBHI.2021.3123657 | Shuo Zhang | 2022 | China | Cohort study(Retrospective) | Single-center study | Acute ischemic stroke |  | 365d | 108 | 2275 | Clinical features | Ten-fold cross-validation | DL |
| 60 | Identification of key predictors of hospital mortality in critically ill patients with embolic stroke using machine learning | 35993194 | <https://doi.org/10.1042/BSR20220995> | Wei Liu | 2022 | China | Cohort study(Retrospective) | MIMIC-IV；eICU-CRD | Acute ischemic stroke |  | In-Hospital Mortality | 282 | 1773 | Clinical features | Random division--7：3；External validation(eICU-CRD) | RF;XGBoost;KNN |
| 61 | Machine learning-based prognostication of mortality in stroke patients | 38601648 | https://doi.org/10.1016/j.heliyon.2024.e28869 | Ahmad A. Abujaber | 2024 | Qatar | Cohort study(Retrospective) | Single-center study | Not specific |  | 365d | 511 | 9,840 | Clinical features | Random division--8：2 | XGBoost;RF;SVM;AdaBoost;LR;NB;KNN |
| 62 | Hospital Length of Stay and 30-Day Mortality Prediction in Stroke: A Machine Learning Analysis of 17,000 ICU Admissions in Brazil | 35381967 | https://doi.org/10.1007/s12028-022-01486-3 | Pedro Kurtz | 2022 | Brazil | Cohort study(Retrospective) | Multicenter study | Not specific |  | In-Hospital Mortality | 1,392 | 16,592 | Clinical features | Random division--8：2 | RF;GBM;LR;KNN;DT |
| 63 | Analyzing and predicting the risk of death in stroke patients using machine learning | 36816575 | 10.3389/fneur.2023.1096153 | Enzhao Zhu | 2023 | China | Case-control study | MIMIC-IV | Not specific |  | 30d | 1,414 | 7,483 | Clinical features | Random division--7.5：2.5 | Ensemble learning;XGBoost;ANN; CatBoost;LR |
| 64 | Derivation and Validation of a New Visceral Adiposity Index for Predicting Short-Term Mortality of Patients with Acute Ischemic Stroke in a Chinese Population | 36831840 | 10.3390/brainsci13020297 | Yuhong Chang | 2023 | China | Case-control study | Single-center study | Acute ischemic stroke |  | 90d | 28 | 197 | Clinical features | Five-fold cross-validation | LR |
| 65 | Development and Validation of a Dynamic Nomogram for Predicting 3-Month Mortality in Acute Ischemic Stroke Patients with Atrial Fibrillation | 38250220 | 10.2147/RMHP.S442353 | Xiaodi Yan | 2024 | China | Cohort study(Retrospective) | Single-center study | Acute ischemic stroke |  | 90d | 88 | 412 | Clinical features | Random division--7：3 | LR |
| 66 | Developing and externally validating a machine learning risk prediction model for 30-day mortality after stroke using national stroke registers in the UK and Sweden | 37968001 | http://dx.doi.org/10.1136/ bmjopen-2022-069 | Wenjuan Wang | 2023 | China | Cohort study(Retrospective) | SSNAP；Riksstroke | Not specific |  | In-Hospital Mortality | 74235 | 488,497 | Clinical features | Temporal validation + External validation（Riksstroke） | LR;XGBoost |
| 67 | Risk factors and prediction of very short term versus short/intermediate term post-stroke mortality: A data mining approach | 25303114 | https://doi.org/10.1016/j.compbiomed.2014.09.003 | Jonathan F. Easton | 2014 | UK | Cohort study(Retrospective) | Multicenter study | Not specific |  | 93d | 242 | 933 | Clinical features | Random division--2：1 | NB DT LR |
| 68 | Predictive Model and Mortality Risk Score during Admission for Ischaemic Stroke with Conservative Treatment | 35328867 | 10.3390/ijerph19063182 | María Carmen Lea-Pereira | 2022 | Spain | Case-control study | Minimum Basic Data Set (MBDS) | Acute ischemic stroke |  | In-Hospital Mortality | 12,665 | 186,245 | Clinical features | Random division--8：2 | LR;RF;ANN |

**Table S2.** Process of PROBAS assessment of RoB in included studies mortality in stroke in the validation set.

| No | Author | Year | Populations | | Predictors | | | Outcomes | | | | | | Statistics | | | | | | | | |
| --- | --- | --- | --- | --- | --- | --- | --- | --- | --- | --- | --- | --- | --- | --- | --- | --- | --- | --- | --- | --- | --- | --- |
|  |  |  | 1.1 | 1.2 | 2.1 | 2.2 | 2.3 | question1 | question2 | question3 | question4 | question5 | question6 | 4.1 | 4.2 | 4.3 | 4.4 | 4.5 | 4.6 | 4.7 | 4.8 | 4.9 |
| 40 | O G Solberg [77] | 2007 | 1 | 1 | 1 | 1 | 1 | 1 | 1 | 1 | 1 | 1 | 1 | 1 | 1 | 1 | 2 | 2 | 0 | 1 | 1 | 1 |
| 6 | Süt and Celik [102] | 2012 | 2 | 1 | 1 | 2 | 1 | 1 | 1 | 1 | 1 | 1 | 1 | 1 | 1 | 1 | 1 | 2 | 2 | 1 | 1 | 1 |
| 46 | Jason Lee [83] | 2013 | 2 | 1 | 1 | 2 | 1 | 1 | 1 | 1 | 1 | 1 | 1 | 1 | 1 | 1 | 0 | 2 | 2 | 1 | 1 | 1 |
| 36 | Guner ¸ Celik [73] | 2014 | 2 | 1 | 1 | 2 | 1 | 1 | 1 | 1 | 1 | 1 | 1 | 1 | 1 | 1 | 2 | 1 | 2 | 1 | 1 | 1 |
| 47 | Jin Guangyong [84] | 2014 | 1 | 1 | 1 | 1 | 1 | 1 | 1 | 1 | 1 | 1 | 1 | 1 | 1 | 1 | 1 | 1 | 1 | 1 | 1 | 1 |
| 53 | Benjamin D Bray [29] | 2014 | 1 | 1 | 1 | 1 | 1 | 1 | 1 | 1 | 1 | 1 | 1 | 1 | 1 | 1 | 1 | 1 | 1 | 1 | 1 | 1 |
| 67 | Easton et al [100] | 2014 | 1 | 1 | 1 | 1 | 1 | 1 | 1 | 1 | 1 | 1 | 1 | 1 | 1 | 1 | 1 | 2 | 1 | 1 | 1 | 1 |
| 54 | Jennifer Schwartz [89] | 2017 | 1 | 1 | 1 | 1 | 1 | 1 | 1 | 1 | 1 | 1 | 1 | 1 | 1 | 1 | 1 | 1 | 1 | 1 | 1 | 1 |
| 11 | Eunjeong Park [49] | 2018 | 1 | 1 | 1 | 1 | 1 | 1 | 1 | 1 | 1 | 1 | 1 | 1 | 1 | 1 | 1 | 1 | 1 | 1 | 2 | 1 |
| 49 | Gattringer et al [103] | 2018 | 1 | 1 | 1 | 1 | 1 | 1 | 1 | 1 | 1 | 1 | 1 | 1 | 1 | 1 | 2 | 1 | 1 | 1 | 1 | 1 |
| 50 | Gao et al [86] | 2018 | 1 | 1 | 1 | 1 | 1 | 1 | 1 | 1 | 1 | 1 | 1 | 1 | 1 | 1 | 2 | 1 | 1 | 1 | 1 | 1 |
| 52 | Cappellari Manuel et al [88] | 2018 | 1 | 1 | 1 | 1 | 1 | 1 | 1 | 1 | 1 | 1 | 1 | 2 | 1 | 1 | 1 | 2 | 2 | 1 | 1 | 1 |
| 39 | Wenzhe Sun [76] | 2019 | 2 | 1 | 1 | 2 | 1 | 1 | 1 | 1 | 1 | 1 | 1 | 1 | 1 | 1 | 1 | 2 | 0 | 1 | 2 | 1 |
| 8 | Domenico Scrutinio [46] | 2020 | 1 | 1 | 1 | 1 | 1 | 1 | 1 | 1 | 1 | 1 | 1 | 1 | 1 | 1 | 1 | 1 | 1 | 1 | 1 | 1 |
| 32 | Xiaohao Zhang [69] | 2020 | 1 | 1 | 1 | 1 | 1 | 1 | 1 | 1 | 1 | 1 | 1 | 1 | 1 | 1 | 1 | 1 | 1 | 1 | 1 | 1 |
| 41 | Ling Sha [78] | 2020 | 1 | 1 | 1 | 1 | 1 | 1 | 1 | 1 | 1 | 1 | 1 | 1 | 1 | 1 | 1 | 1 | 1 | 1 | 1 | 1 |
| 43 | J. Liljehult [80] | 2020 | 1 | 1 | 1 | 1 | 1 | 1 | 1 | 1 | 1 | 1 | 1 | 1 | 1 | 1 | 2 | 1 | 1 | 1 | 1 | 1 |
| 57 | Huang et al [92] | 2020 | 1 | 1 | 1 | 1 | 1 | 1 | 1 | 1 | 1 | 1 | 1 | 1 | 1 | 1 | 1 | 1 | 1 | 1 | 1 | 1 |
| 23 | Carlos Fernandez-Lozano [60] | 2021 | 1 | 1 | 1 | 1 | 1 | 1 | 1 | 1 | 1 | 1 | 1 | 1 | 1 | 1 | 2 | 2 | 2 | 1 | 1 | 1 |
| 4 | Wenjuan Wang [43] | 2022 | 1 | 1 | 1 | 1 | 1 | 1 | 1 | 1 | 1 | 1 | 1 | 1 | 1 | 1 | 1 | 1 | 1 | 1 | 1 | 1 |
| 15 | Lehmann et al [53] | 2022 | 1 | 1 | 1 | 1 | 1 | 1 | 1 | 1 | 1 | 1 | 1 | 2 | 1 | 1 | 2 | 1 | 1 | 1 | 1 | 1 |
| 16 | Helge C. Kniep [28] | 2022 | 1 | 1 | 1 | 1 | 1 | 1 | 1 | 1 | 1 | 1 | 1 | 2 | 1 | 1 | 2 | 1 | 1 | 1 | 1 | 1 |
| 30 | Vida Abedi [67] | 2022 | 1 | 1 | 1 | 1 | 1 | 1 | 1 | 1 | 1 | 1 | 1 | 1 | 1 | 1 | 1 | 1 | 1 | 1 | 1 | 1 |
| 33 | Rui-xin Zhang [70] | 2022 | 2 | 1 | 1 | 2 | 1 | 1 | 1 | 1 | 1 | 1 | 1 | 2 | 1 | 1 | 2 | 1 | 1 | 1 | 1 | 1 |
| 38 | Weronika A Szlachetka [75] | 2022 | 1 | 1 | 1 | 1 | 1 | 1 | 1 | 1 | 1 | 1 | 1 | 1 | 1 | 1 | 1 | 1 | 1 | 1 | 1 | 1 |
| 44 | Xiao-Dan Li [81] | 2022 | 1 | 1 | 1 | 1 | 1 | 1 | 1 | 1 | 1 | 1 | 1 | 1 | 1 | 1 | 2 | 2 | 1 | 1 | 1 | 1 |
| 56 | Lee et al [91] | 2022 | 1 | 1 | 1 | 1 | 1 | 1 | 1 | 1 | 1 | 1 | 1 | 1 | 1 | 1 | 2 | 1 | 2 | 1 | 1 | 1 |
| 60 | Liu et al [26] | 2022 | 1 | 1 | 1 | 1 | 1 | 1 | 1 | 1 | 1 | 1 | 1 | 1 | 1 | 1 | 1 | 1 | 1 | 1 | 1 | 1 |
| 59 | Zhang et al [94] | 2022 | 1 | 1 | 1 | 1 | 1 | 1 | 1 | 1 | 1 | 1 | 1 | 1 | 1 | 1 | 2 | 1 | 1 | 1 | 1 | 1 |
| 62 | Pedro Kurtz [27] | 2022 | 1 | 1 | 1 | 1 | 1 | 1 | 1 | 1 | 1 | 1 | 1 | 1 | 1 | 1 | 1 | 1 | 1 | 1 | 1 | 1 |
| 68 | María Carmen Lea-Pereira et al [101] | 2022 | 1 | 1 | 1 | 1 | 1 | 1 | 1 | 1 | 1 | 1 | 1 | 1 | 1 | 1 | 1 | 1 | 1 | 1 | 1 | 1 |
| 5 | Kai Wang [44] | 2023 | 1 | 1 | 1 | 1 | 1 | 1 | 1 | 1 | 1 | 1 | 1 | 2 | 1 | 1 | 1 | 1 | 1 | 1 | 1 | 1 |
| 7 | Nasrin Someeh [45] | 2023 | 1 | 1 | 1 | 1 | 1 | 1 | 1 | 1 | 1 | 1 | 1 | 1 | 1 | 1 | 1 | 1 | 1 | 1 | 1 | 1 |
| 12 | Yang Ouyang [50] | 2023 | 1 | 1 | 1 | 1 | 1 | 1 | 1 | 1 | 1 | 1 | 1 | 1 | 1 | 1 | 1 | 1 | 1 | 1 | 1 | 1 |
| 22 | Haydn Hoffman [59] | 2024 | 1 | 1 | 1 | 1 | 1 | 1 | 1 | 1 | 1 | 1 | 1 | 1 | 1 | 1 | 1 | 1 | 1 | 1 | 1 | 1 |
| 21 | Ruixuan Huang [55] | 2023 | 1 | 1 | 1 | 1 | 1 | 1 | 1 | 1 | 1 | 1 | 1 | 1 | 1 | 1 | 1 | 1 | 1 | 1 | 1 | 1 |
| 20 | Huang et al [57] | 2023 | 1 | 1 | 1 | 1 | 1 | 1 | 1 | 1 | 1 | 1 | 1 | 1 | 1 | 1 | 1 | 1 | 1 | 1 | 1 | 1 |
| 21 | Jian Huang [58] | 2023 | 1 | 1 | 1 | 1 | 1 | 1 | 1 | 1 | 1 | 1 | 1 | 1 | 1 | 1 | 1 | 1 | 1 | 1 | 1 | 1 |
| 28 | Che Muhammad Nur Hidayat Che Nawi [65] | 2023 | 1 | 1 | 1 | 1 | 1 | 1 | 1 | 1 | 1 | 1 | 1 | 1 | 1 | 1 | 1 | 2 | 1 | 1 | 1 | 1 |
| 48 | Jin et al [85] | 2023 | 1 | 1 | 1 | 1 | 1 | 1 | 1 | 1 | 1 | 1 | 1 | 1 | 1 | 1 | 1 | 1 | 1 | 1 | 1 | 1 |
| 63 | Zhu et al [96] | 2023 | 1 | 1 | 1 | 1 | 1 | 1 | 1 | 1 | 1 | 1 | 1 | 1 | 1 | 1 | 2 | 1 | 2 | 1 | 1 | 1 |
| 64 | Chang et al [97] | 2023 | 2 | 1 | 1 | 2 | 1 | 1 | 1 | 1 | 1 | 1 | 1 | 2 | 1 | 1 | 0 | 2 | 2 | 1 | 1 | 1 |
| 66 | Wang et al [99] | 2023 | 1 | 1 | 1 | 1 | 1 | 1 | 1 | 1 | 1 | 1 | 1 | 1 | 1 | 1 | 1 | 1 | 1 | 1 | 1 | 1 |
| 3 | Lingyu Xu [42] | 2024 | 1 | 1 | 1 | 1 | 1 | 1 | 1 | 1 | 1 | 1 | 1 | 1 | 1 | 1 | 2 | 1 | 1 | 1 | 1 | 1 |
| 9 | Ivan Petrović [47] | 2024 | 2 | 1 | 1 | 2 | 1 | 1 | 1 | 1 | 1 | 1 | 1 | 2 | 1 | 1 | 1 | 2 | 1 | 1 | 1 | 1 |
| 14 | Ton Duy Mai [52] | 2024 | 1 | 1 | 1 | 1 | 1 | 1 | 1 | 1 | 1 | 1 | 1 | 1 | 1 | 1 | 2 | 2 | 2 | 1 | 1 | 1 |
| 17 | Changi Kim [54] | 2024 | 1 | 1 | 1 | 1 | 1 | 1 | 1 | 1 | 1 | 1 | 1 | 1 | 1 | 1 | 2 | 1 | 1 | 1 | 1 | 1 |
| 19 | Jian Huang [56] | 2024 | 1 | 1 | 1 | 1 | 1 | 1 | 1 | 1 | 1 | 1 | 1 | 1 | 1 | 1 | 1 | 1 | 1 | 1 | 1 | 1 |
| 24 | J. Fang [61] | 2024 | 1 | 1 | 1 | 1 | 1 | 1 | 1 | 1 | 1 | 1 | 1 | 1 | 1 | 1 | 1 | 1 | 1 | 1 | 1 | 1 |
| 26 | Chen Yang [63] | 2024 | 1 | 1 | 1 | 1 | 1 | 1 | 1 | 1 | 1 | 1 | 1 | 1 | 1 | 1 | 1 | 1 | 1 | 1 | 1 | 1 |
| 27 | Haoran Chen [64] | 2024 | 1 | 1 | 1 | 1 | 1 | 1 | 1 | 1 | 1 | 1 | 1 | 1 | 1 | 1 | 1 | 1 | 1 | 1 | 1 | 1 |
| 29 | Ahmad Abujaber [66] | 2024 | 1 | 1 | 1 | 1 | 1 | 1 | 1 | 1 | 1 | 1 | 1 | 1 | 1 | 1 | 1 | 1 | 1 | 1 | 1 | 1 |
| 31 | Li Zhou [68] | 2024 | 2 | 1 | 1 | 2 | 1 | 1 | 1 | 1 | 1 | 1 | 1 | 2 | 1 | 1 | 2 | 2 | 1 | 1 | 1 | 1 |
| 35 | Minyan Zeng [72] | 2024 | 1 | 1 | 1 | 1 | 1 | 1 | 1 | 1 | 1 | 1 | 1 | 1 | 1 | 1 | 1 | 1 | 1 | 1 | 1 | 1 |
| 37 | Rui Wen [74] | 2024 | 1 | 1 | 1 | 1 | 1 | 1 | 1 | 1 | 1 | 1 | 1 | 1 | 1 | 1 | 1 | 1 | 1 | 1 | 1 | 1 |
| 42 | Heng Ni [79] | 2024 | 1 | 1 | 1 | 1 | 1 | 1 | 1 | 1 | 1 | 1 | 1 | 2 | 1 | 1 | 2 | 1 | 1 | 1 | 1 | 1 |
| 51 | Lingyan Fang [87] | 2024 | 1 | 1 | 1 | 1 | 1 | 1 | 1 | 1 | 1 | 1 | 1 | 1 | 1 | 1 | 1 | 1 | 1 | 1 | 1 | 1 |
| 61 | Abujaber et al [95] | 2024 | 1 | 1 | 1 | 1 | 1 | 1 | 1 | 1 | 1 | 1 | 1 | 1 | 1 | 1 | 1 | 1 | 1 | 1 | 1 | 1 |
| 65 | Yan et al [98] | 2024 | 1 | 1 | 1 | 1 | 1 | 1 | 1 | 1 | 1 | 1 | 1 | 1 | 1 | 1 | 1 | 1 | 1 | 1 | 1 | 1 |
| 1 | Solmaz Norouzi [40] | 2025 | 1 | 1 | 1 | 1 | 1 | 1 | 1 | 1 | 1 | 1 | 1 | 2 | 1 | 1 | 0 | 1 | 1 | 1 | 1 | 1 |
| 2 | Zhou Zhou [41] | 2025 | 1 | 1 | 1 | 1 | 1 | 1 | 1 | 1 | 1 | 1 | 1 | 2 | 1 | 1 | 1 | 1 | 1 | 1 | 1 | 1 |
| 10 | Jack A Cummins [48] | 2025 | 1 | 1 | 1 | 1 | 1 | 1 | 1 | 1 | 1 | 1 | 1 | 1 | 1 | 1 | 2 | 1 | 1 | 1 | 1 | 1 |
| 13 | Luo Xiao [51] | 2025 | 1 | 1 | 1 | 1 | 1 | 1 | 1 | 1 | 1 | 1 | 1 | 1 | 1 | 1 | 1 | 1 | 1 | 1 | 1 | 1 |
| 25 | Zhang Chunjuan [62] | 2025 | 1 | 1 | 1 | 1 | 1 | 1 | 1 | 1 | 1 | 1 | 1 | 1 | 1 | 1 | 1 | 1 | 2 | 1 | 1 | 1 |
| 34 | Jie Zhang [71] | 2025 | 1 | 1 | 1 | 1 | 1 | 1 | 1 | 1 | 1 | 1 | 1 | 1 | 1 | 1 | 1 | 1 | 1 | 1 | 1 | 1 |
| 45 | Tae Jung Kim [82] | 2025 | 1 | 1 | 1 | 1 | 1 | 1 | 1 | 1 | 1 | 1 | 1 | 1 | 1 | 1 | 2 | 1 | 1 | 1 | 1 | 1 |
| 55 | Lukas Reitzle [90] | 2025 | 1 | 1 | 1 | 1 | 1 | 1 | 1 | 1 | 1 | 1 | 1 | 1 | 1 | 1 | 2 | 1 | 0 | 1 | 1 | 1 |
| 58 | Geng et al [93] | 2025 | 1 | 1 | 1 | 1 | 1 | 1 | 1 | 1 | 1 | 1 | 1 | 1 | 1 | 1 | 1 | 1 | 1 | 1 | 2 | 1 |

**
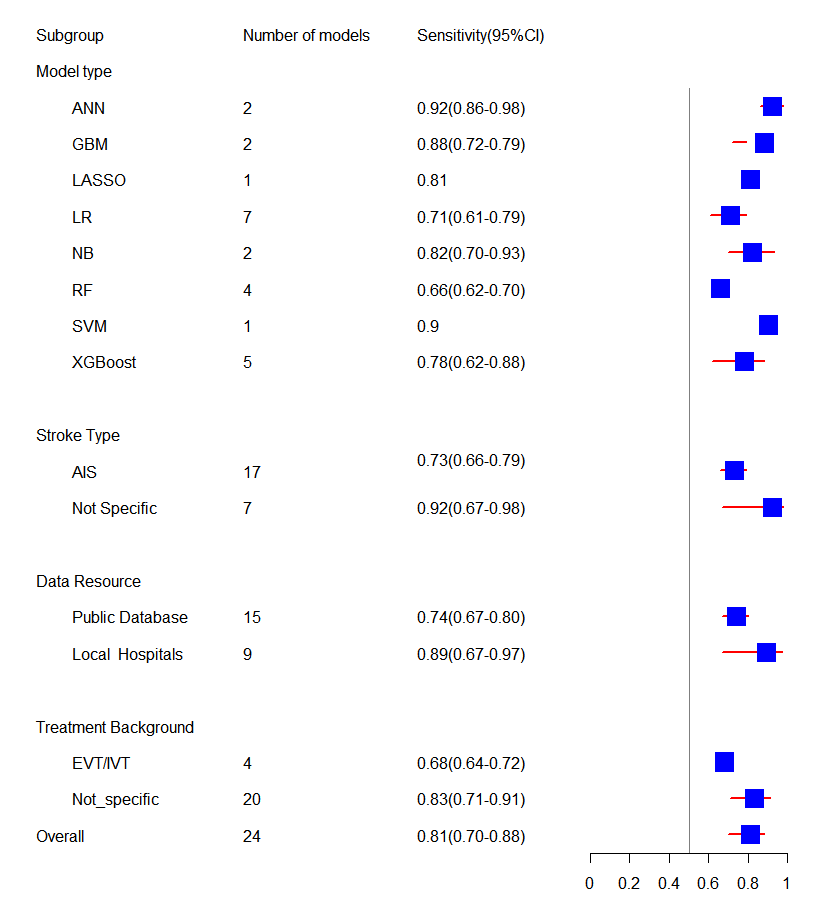
**

**Figure S1.** Forest plot for meta-analysis of SE of ML for predicting in-hospital mortality in stroke in the training set.

**
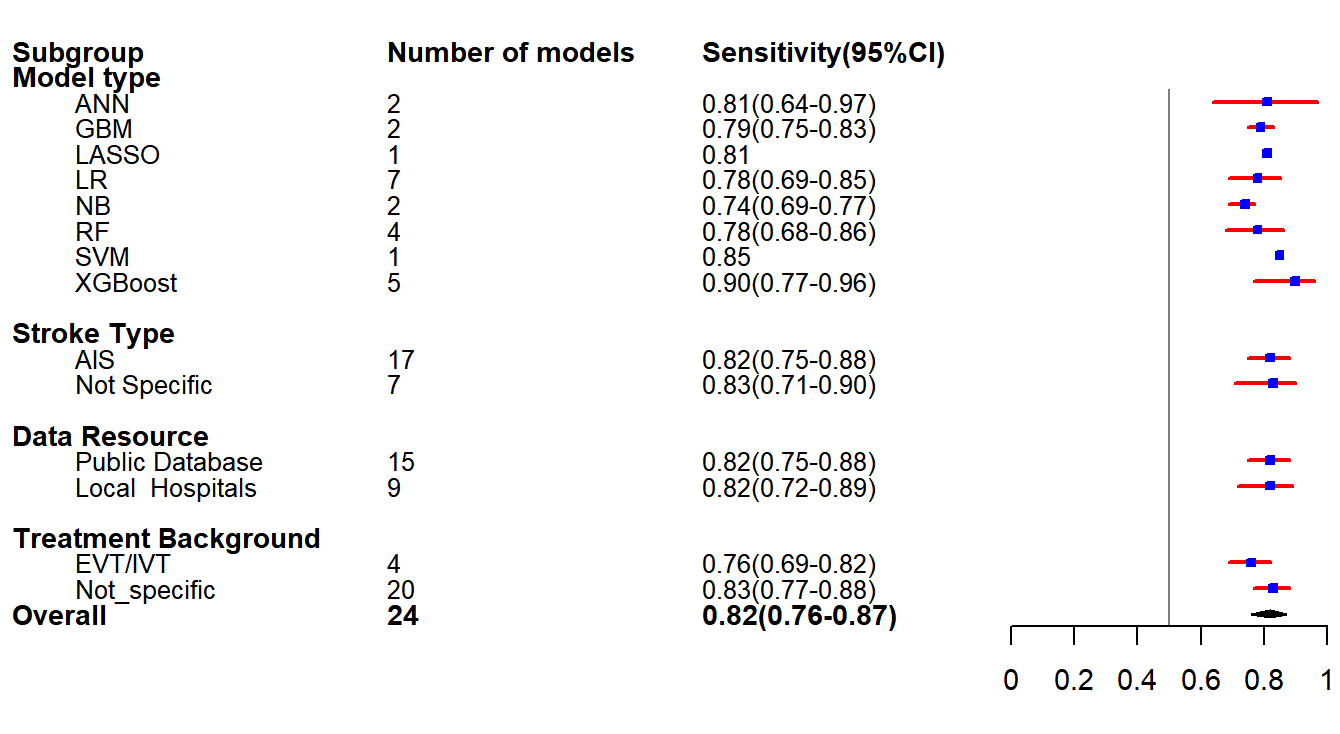
**

**Figure S2.** Forest plot for meta-analysis of SP of ML for predicting in-hospital mortality in stroke in the training set.

**
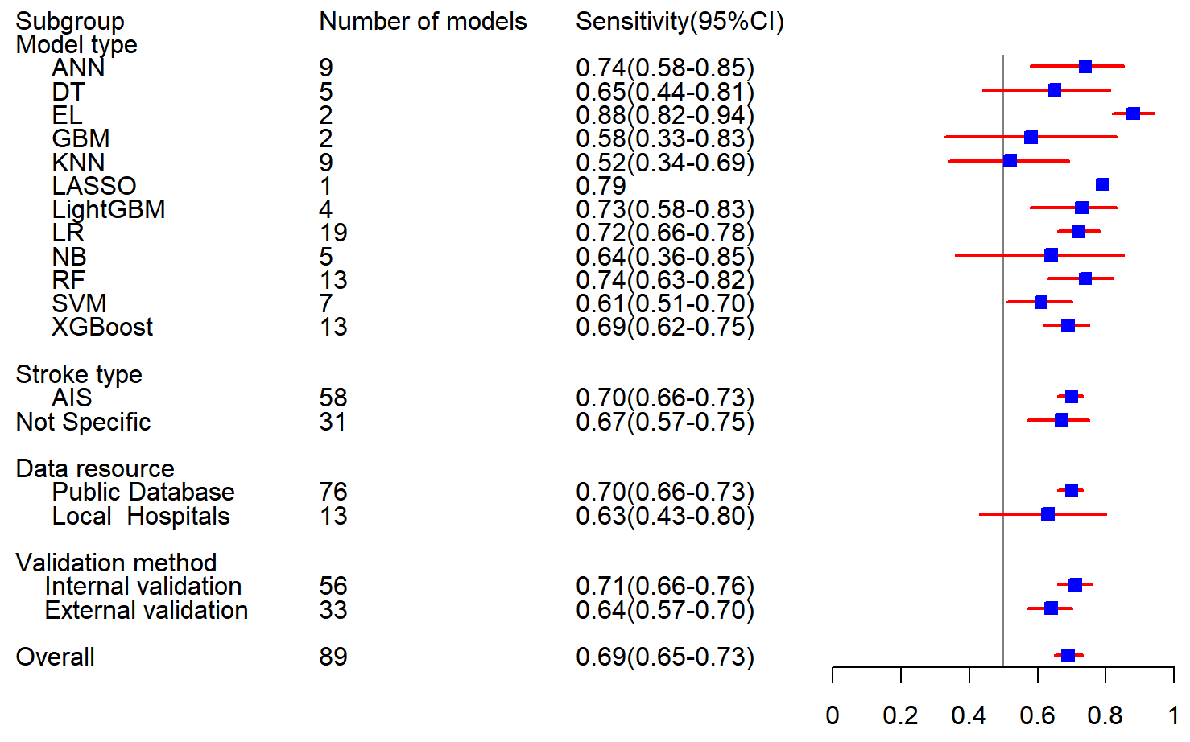
**

**Figure S3.** Forest plot for meta-analysis of SE of ML for predicting in-hospital mortality in stroke in the validation set.

**
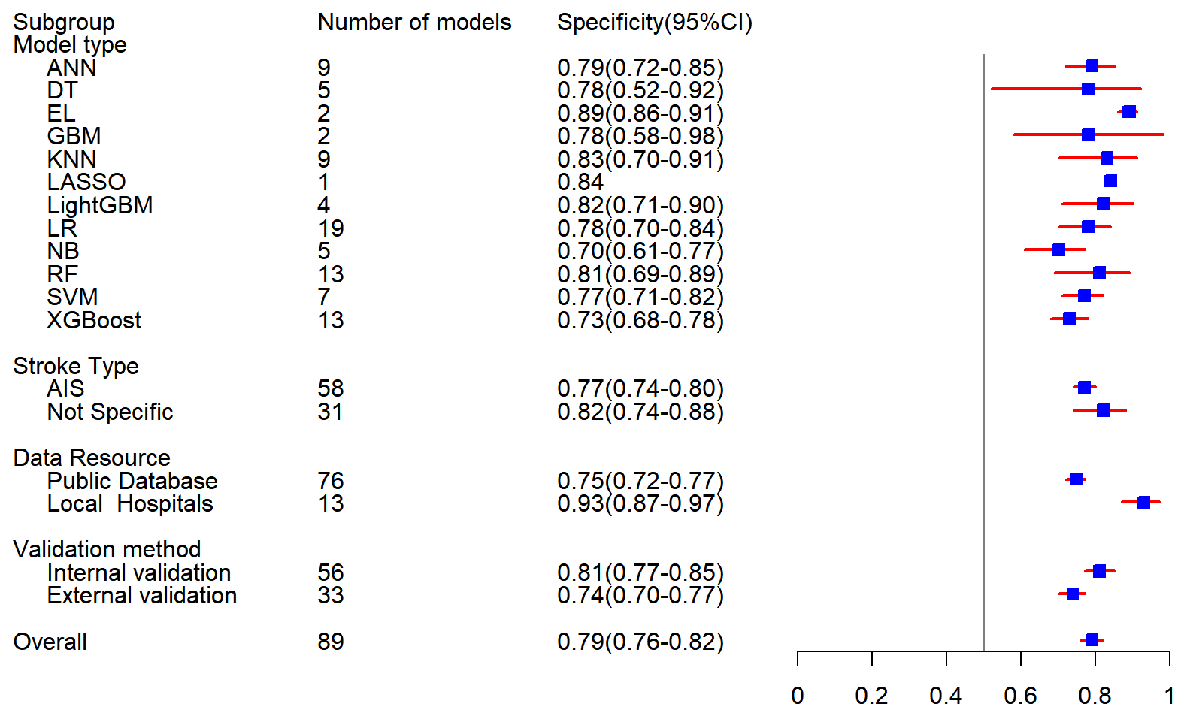
**

**Figure S4.** Forest plot for meta-analysis of SP of ML for predicting in-hospital mortality in stroke in the validation set.

**
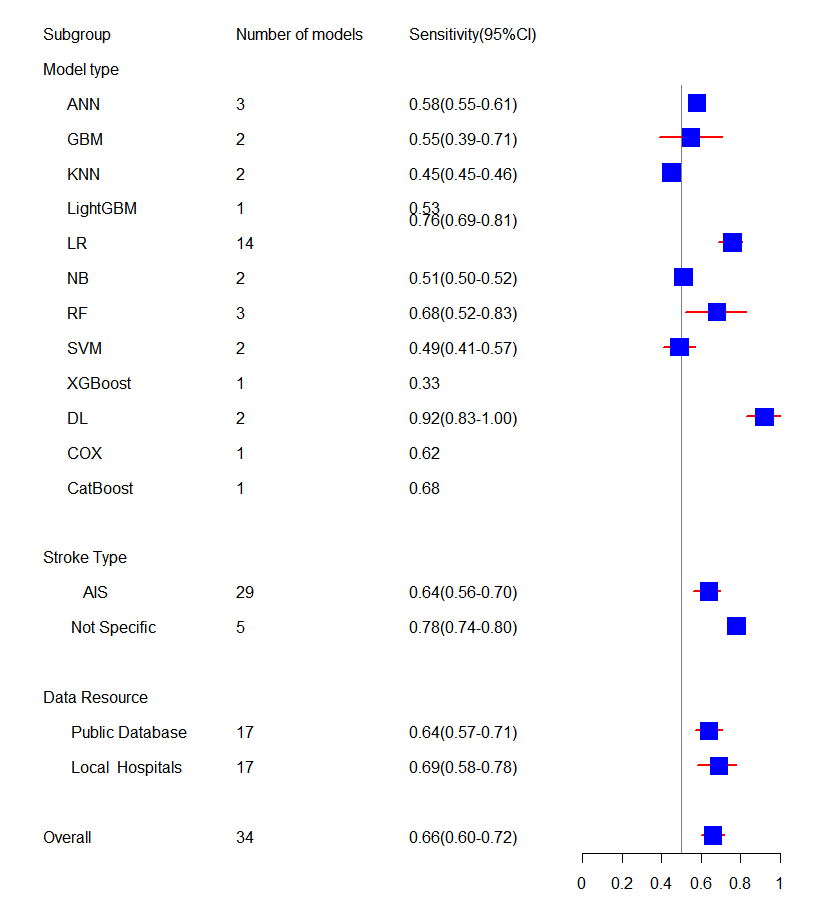
**

**Figure S5.** Forest plot for meta-analysis of SE of ML for predicting out-of-hospital mortality in stroke in the training set.

**
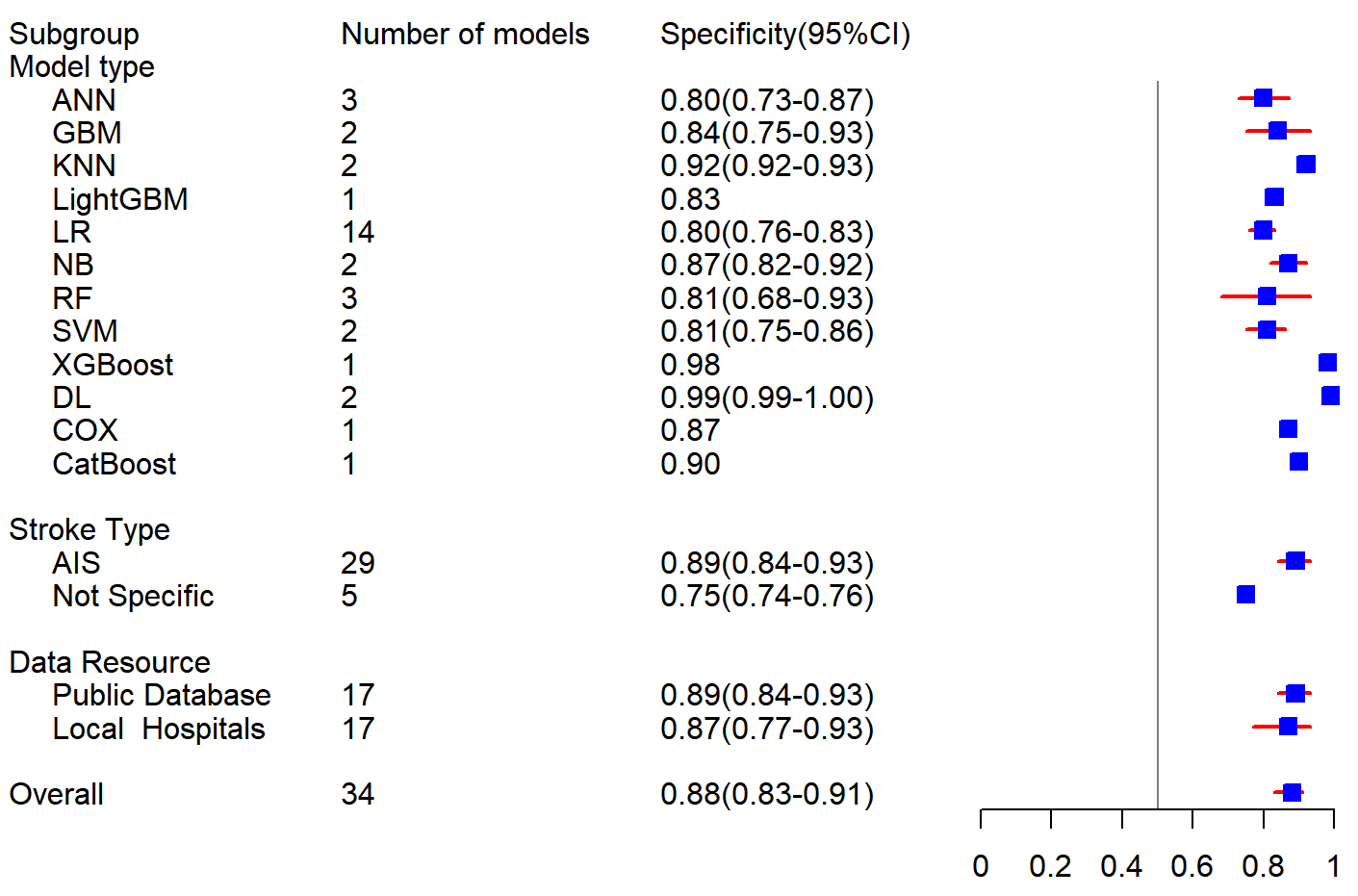
**

**Figure S6.** Forest plot for meta-analysis of SP of ML for predicting out-of-hospital mortality in stroke in the training set.

**
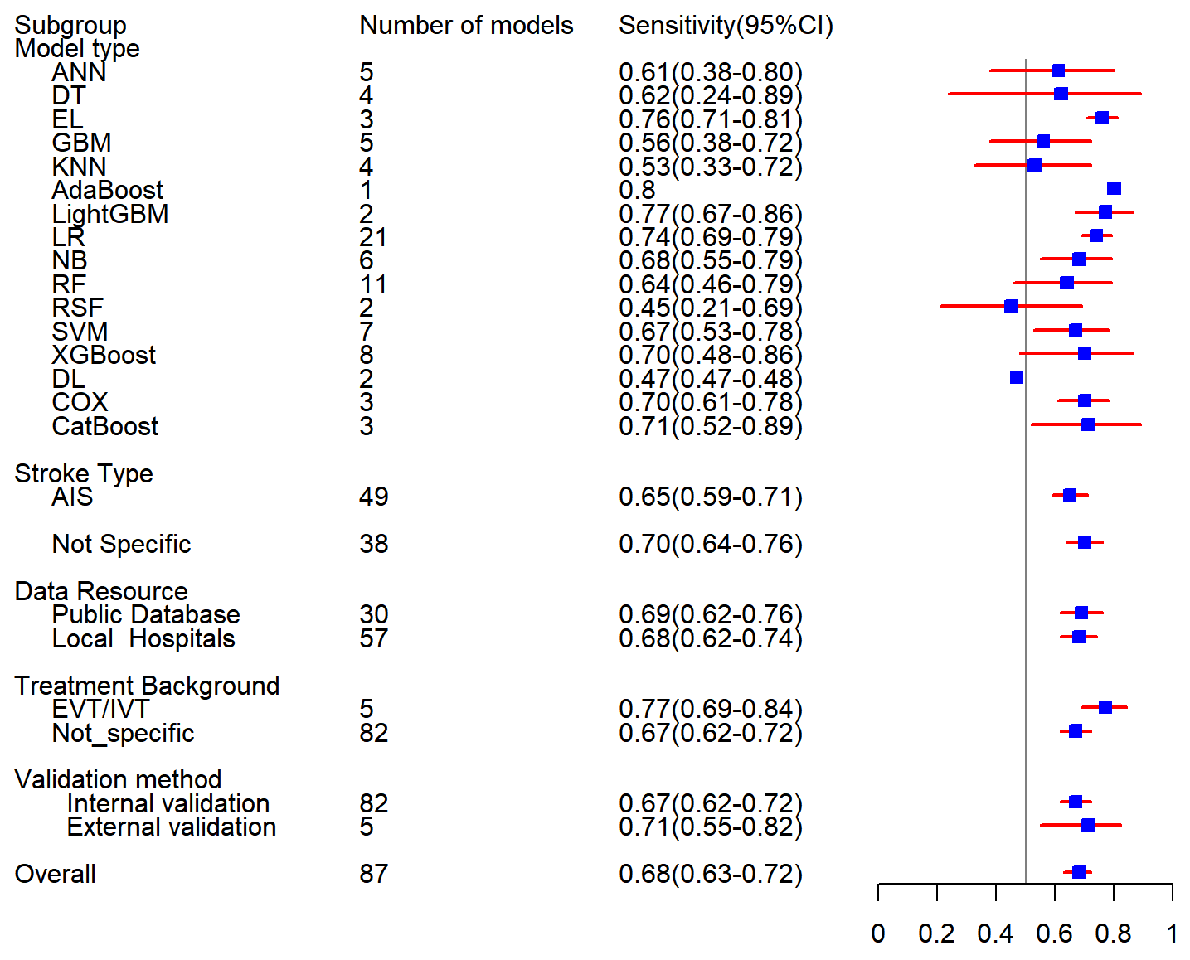
**

**Figure S7.** Forest plot for meta-analysis of SE of ML for predicting out-of-hospital mortality in stroke in the validation set.

**
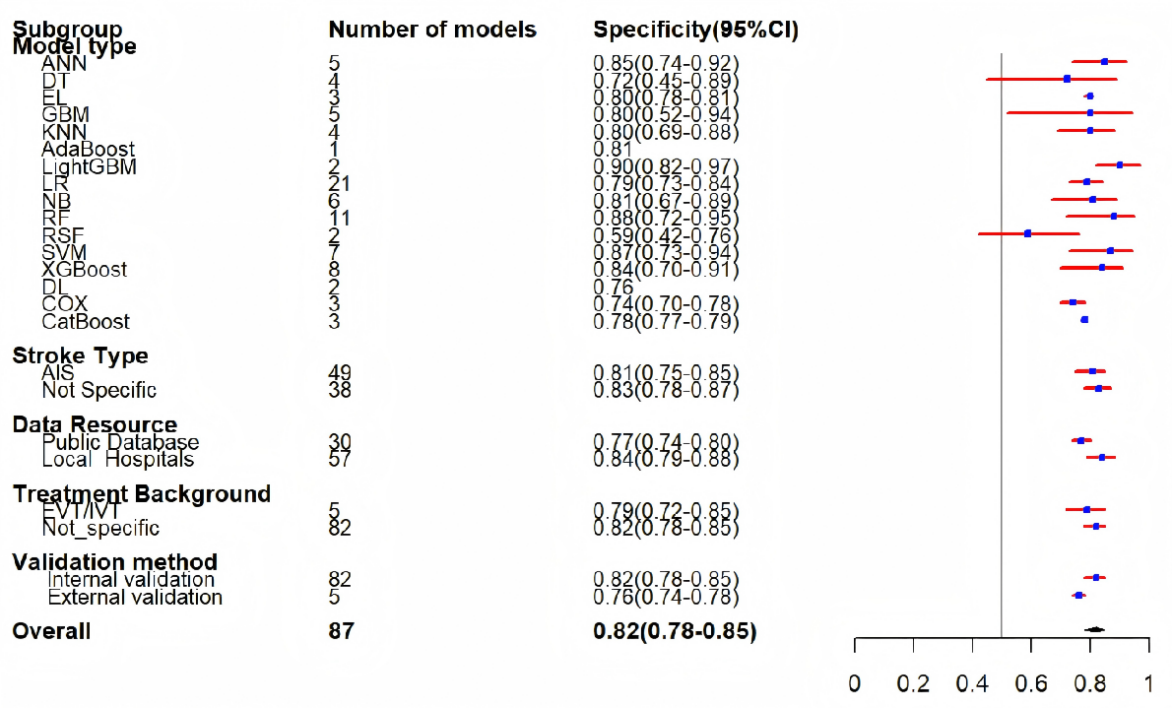
**

**Figure S8.** Forest plot for meta-analysis of SP of ML for predicting out-of-hospital mortality in stroke in the validation set.


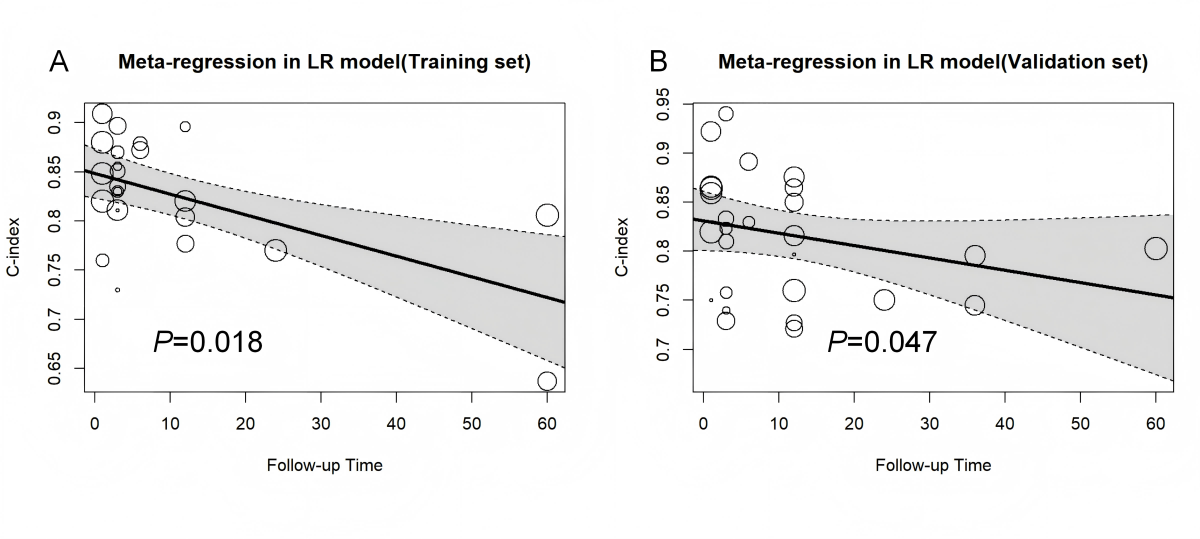


**Figure S9.** Meta-regression bubble plot of follow-up time in the logistic regression model for (A)training set and (B)validation set.


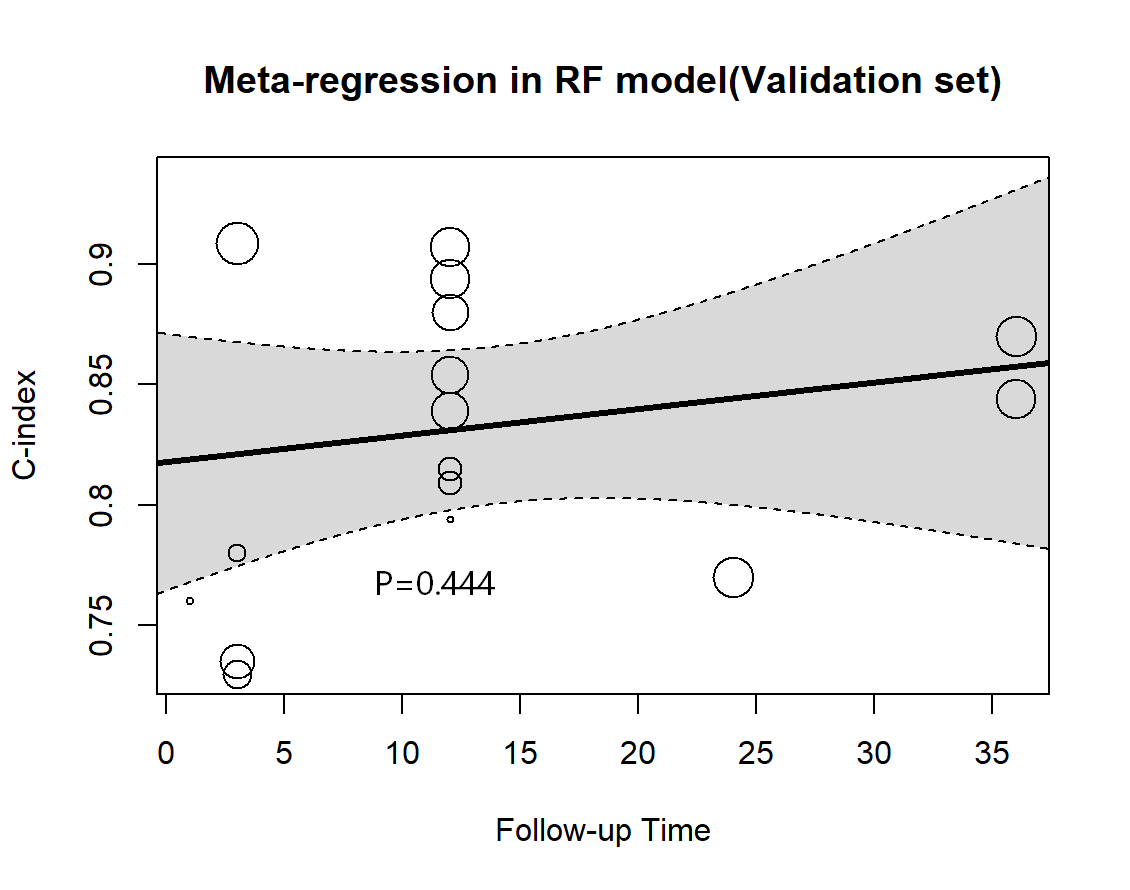


**Figure S10.** Meta-regression bubble plot of follow-up time in the random forest model for (A)training set and (B)validation set.


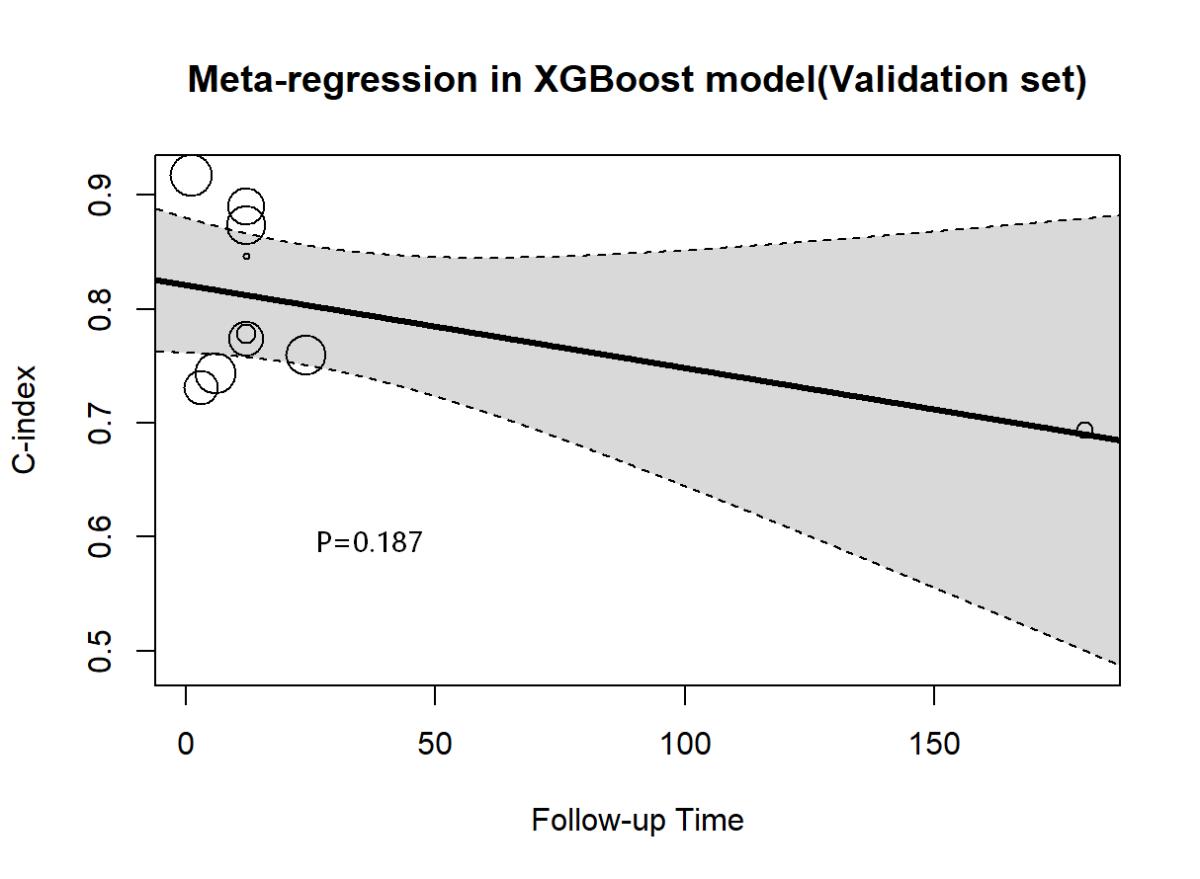


**Figure S11.** Meta-regression bubble plot of follow-up time in the eXtreme Gradient Boosting(XGBoost) model for validation set.


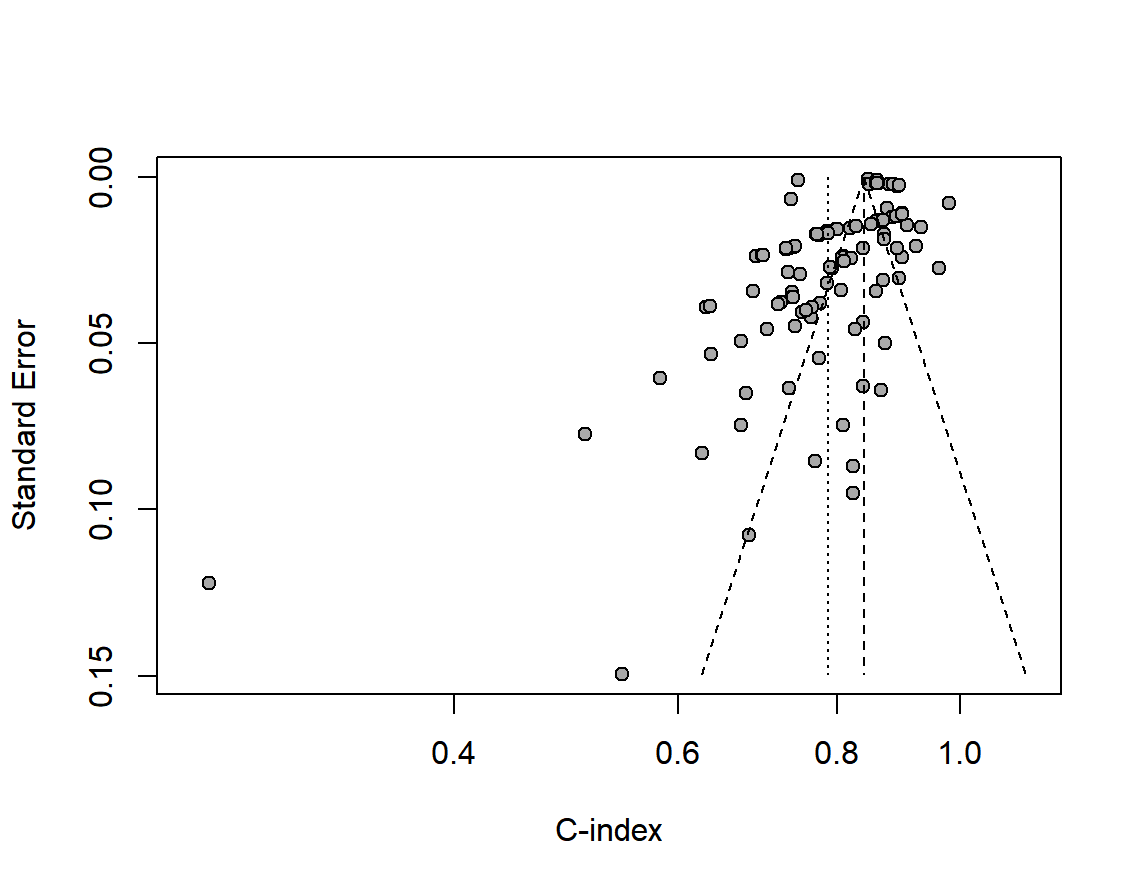


**Figure S12.** Funnel plot of C-index of all predictive models for in-hospital mortality in stroke in the validation set.


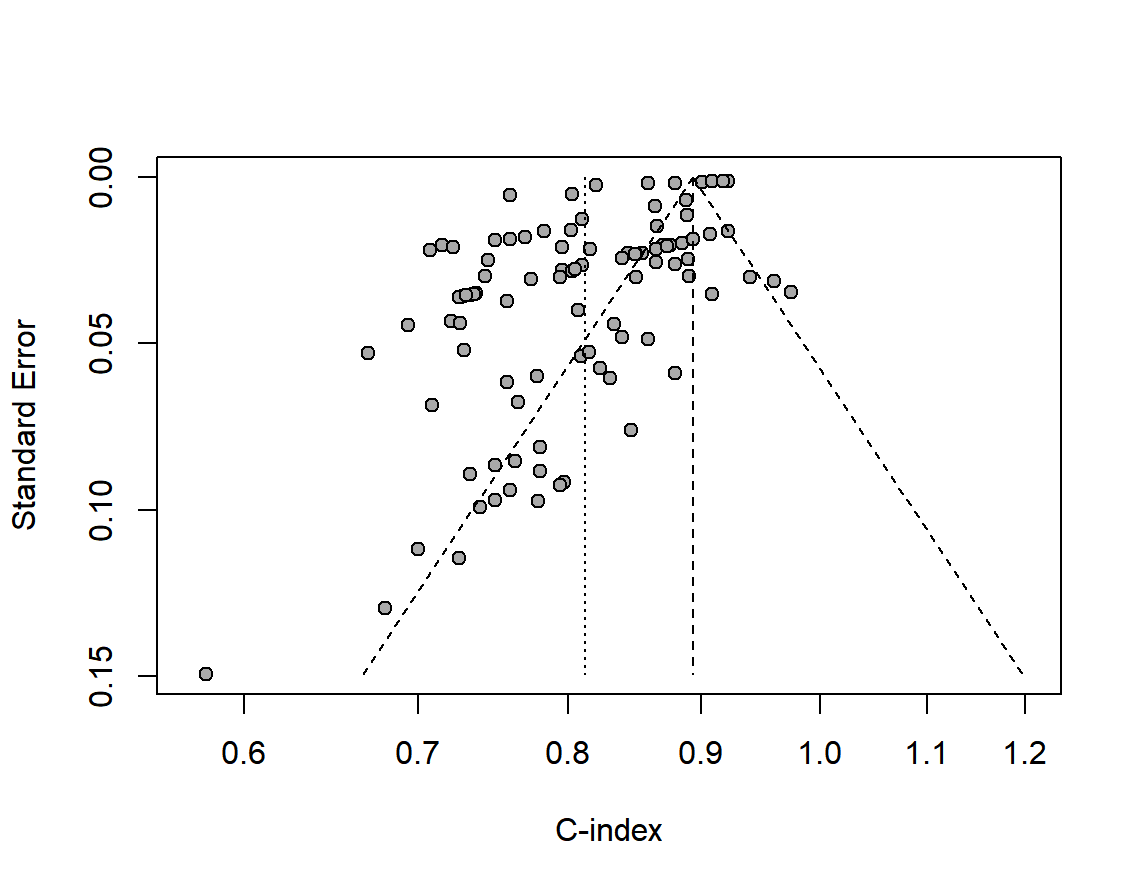


**Figure S13.** Funnel plot of C-index of all predictive models for out-of-hospital mortality in stroke in the validation set.


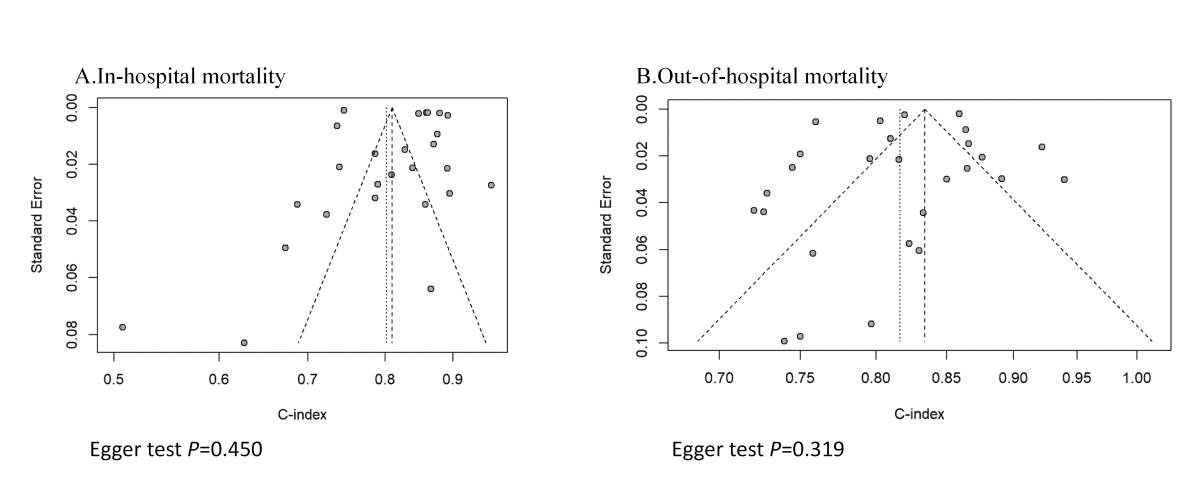


**Figure S14.** Funnel plot of C-index of logistic regression model in stroke in the validation set for (A)in-hospital mortality and (B)out-of-hospital mortality.


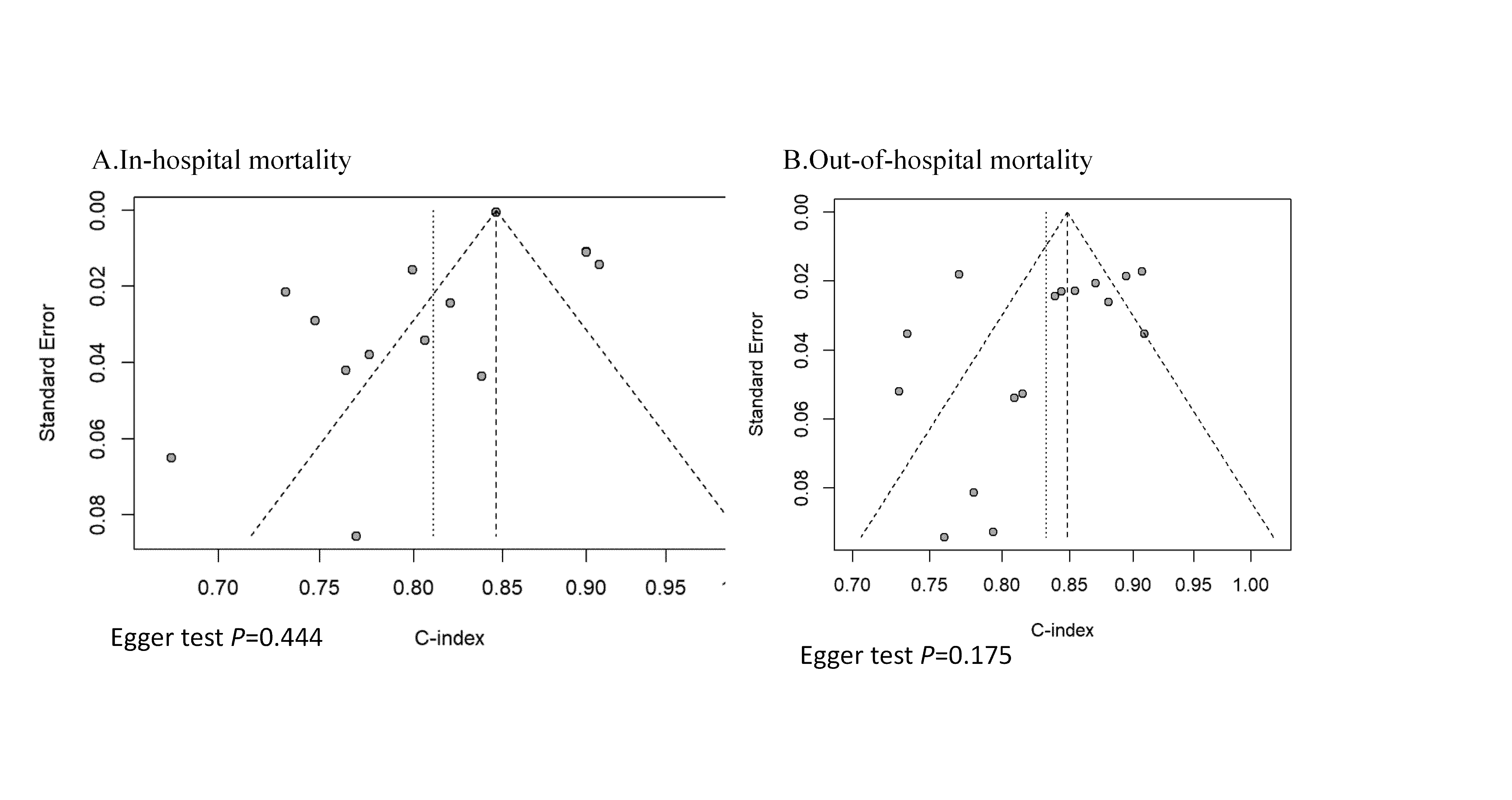


**Figure S15.** Funnel plot of C-index of random forest model in stroke in the validation set for (A)in-hospital mortality and (B)out-of-hospital mortality.


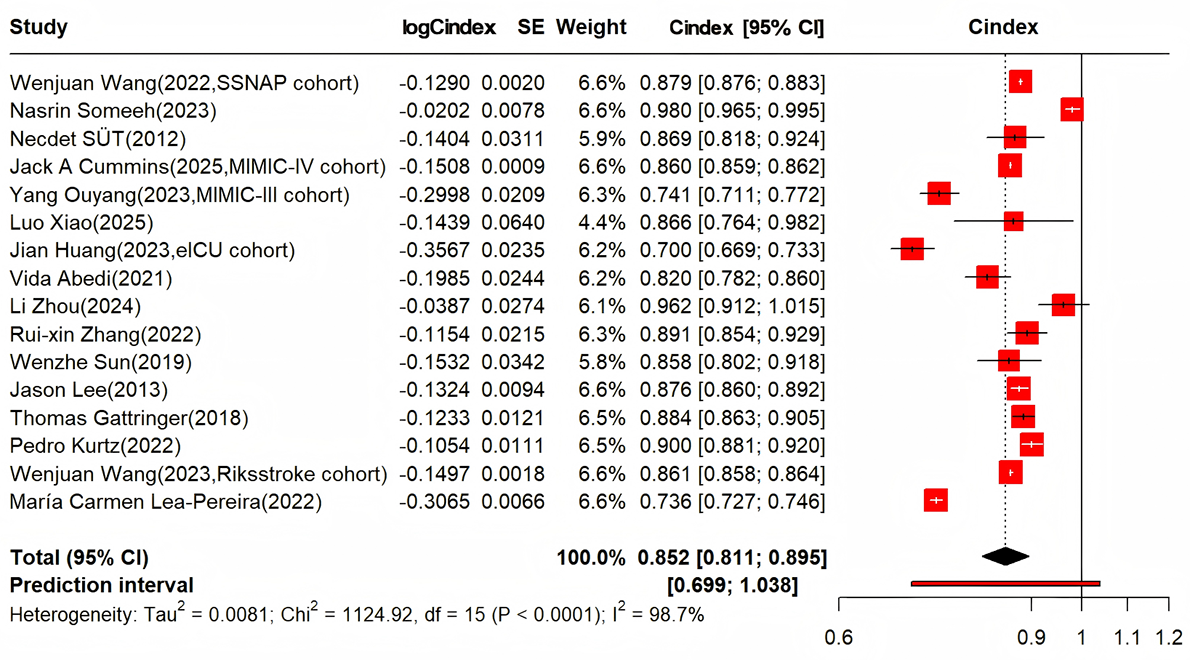


**Figure S16.** Sensitivity.
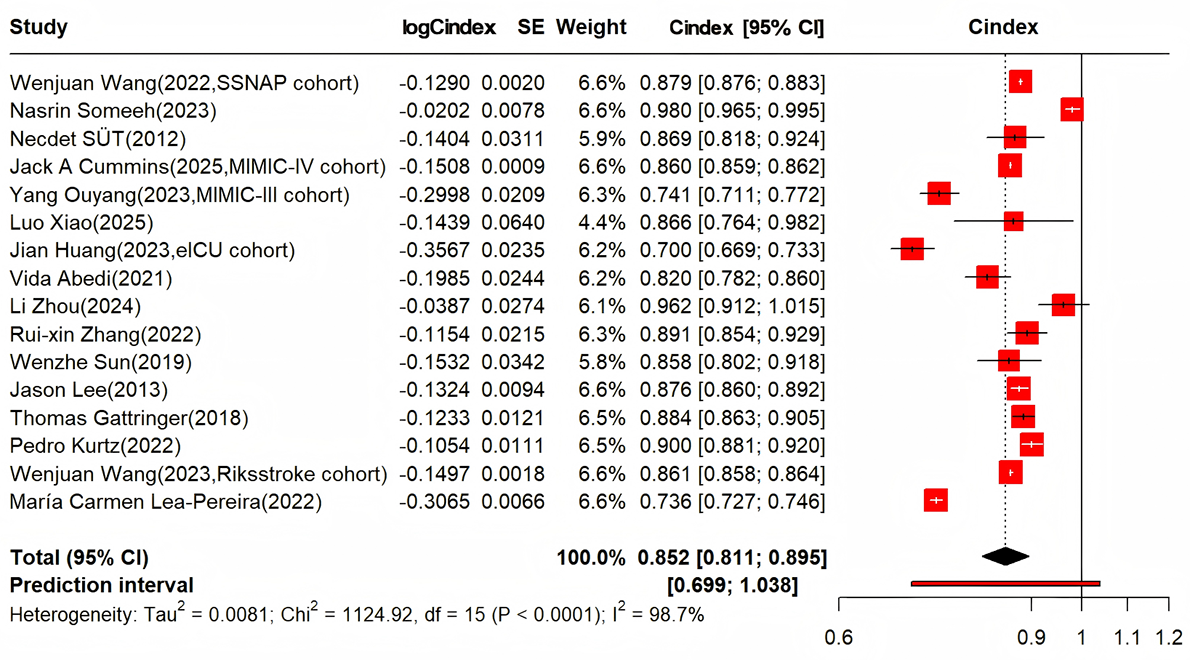
analysis for in-hospital mortality in the validation set [27,43,45,48,50,51,58,67,68,70,76,83,99,101-103].


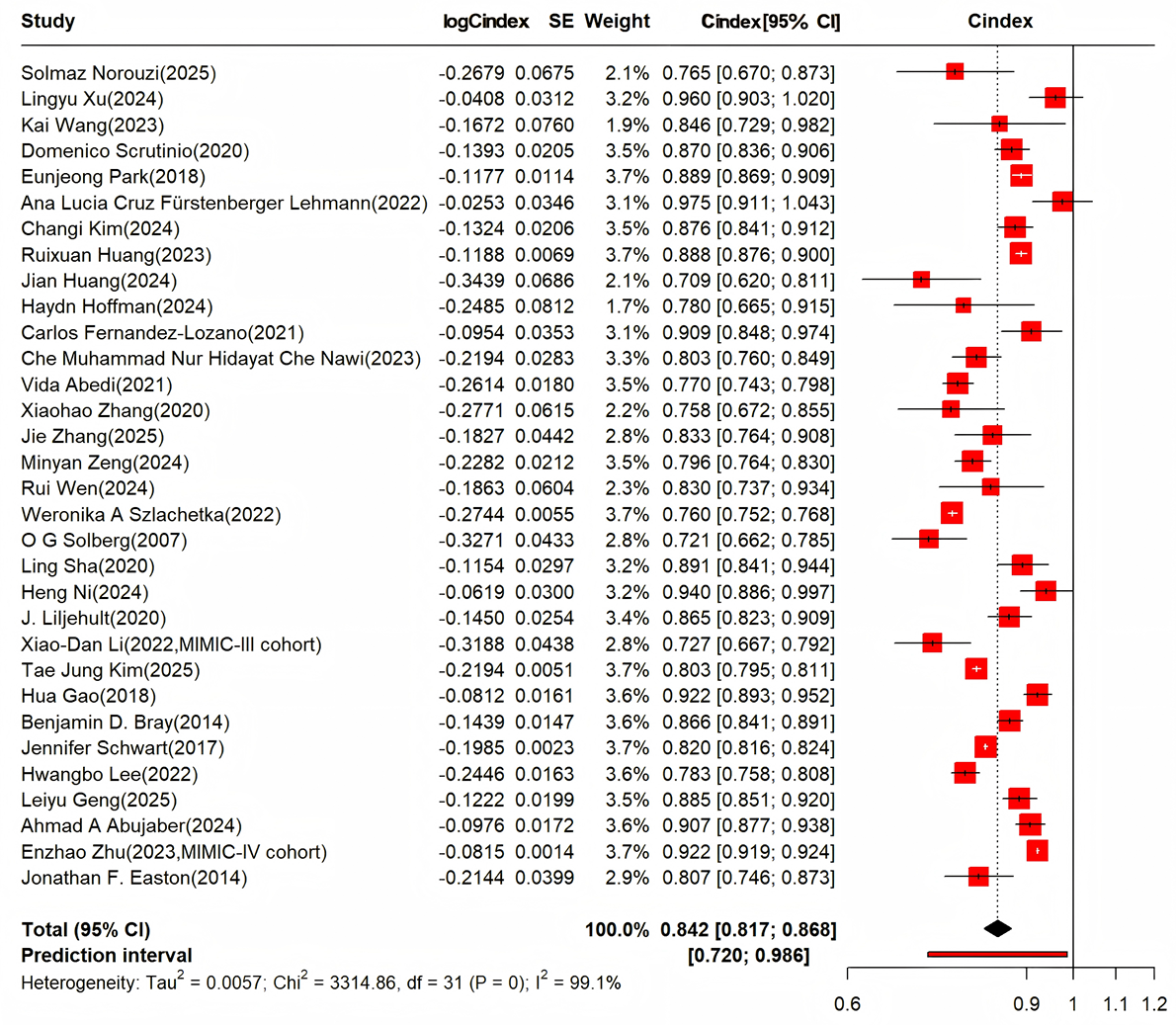


**Figure S17.** Sensitivity analysis for out-of-hospital mortality in the validation set [29,40,42,44,46,49,53-56,59,60,65,67,69,71,72,74,75,77-82,86,89,91,93,95,96,100].


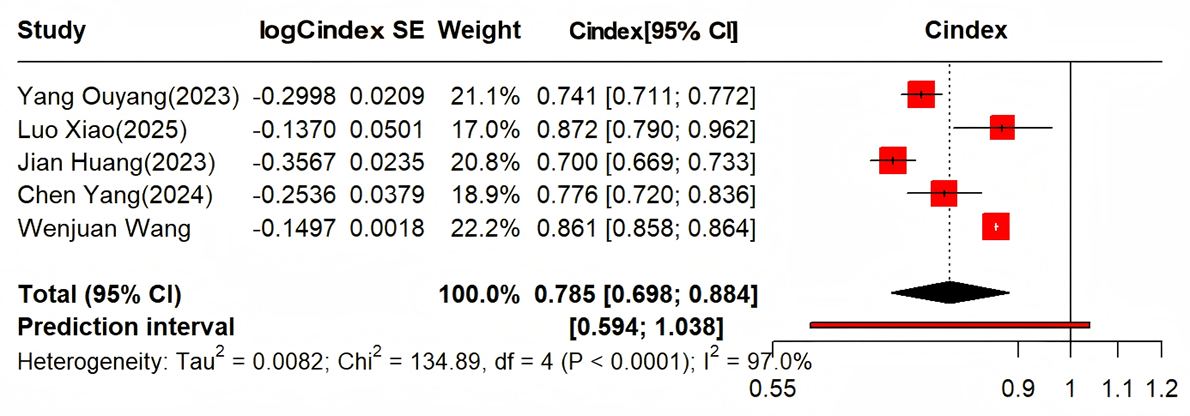


**Figure S18.** Forest plots of the C-index of performance of the model in the external validation for predicting in-of-hospital mortality [50,51,53,63,99].


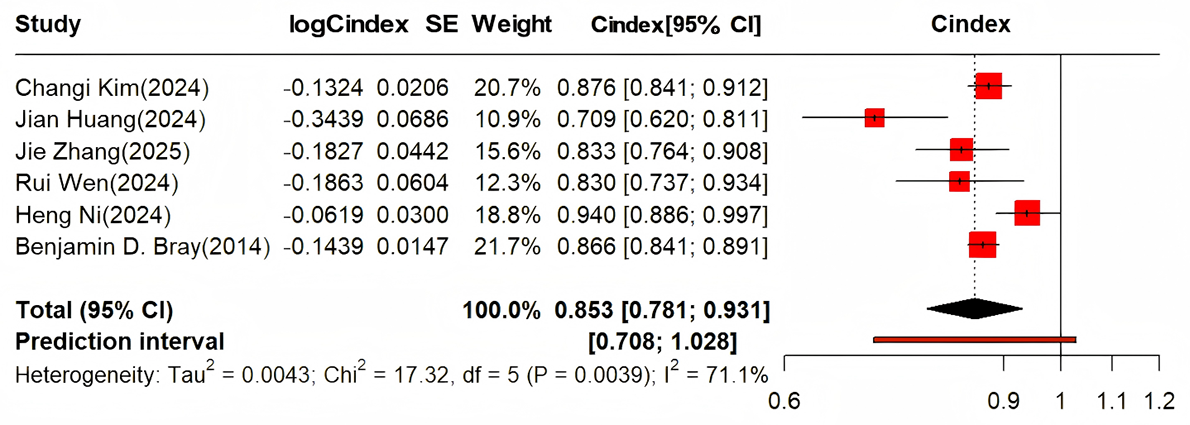


**Figure S19.** Forest plots of the C-index of performance of the model in the external validation for predicting out-of-hospital mortality [29,54,56,71,74,79].
