# Supplementary figures and images for: Predictive Value of Machine Learning for Poststroke Mortality Risk: Systematic Review and Meta-Analysis
Source: J Med Internet Res. 2026 Apr 2;28:e83821. doi: 10.2196/83821 (PMC13087561; doi:10.2196/83821)

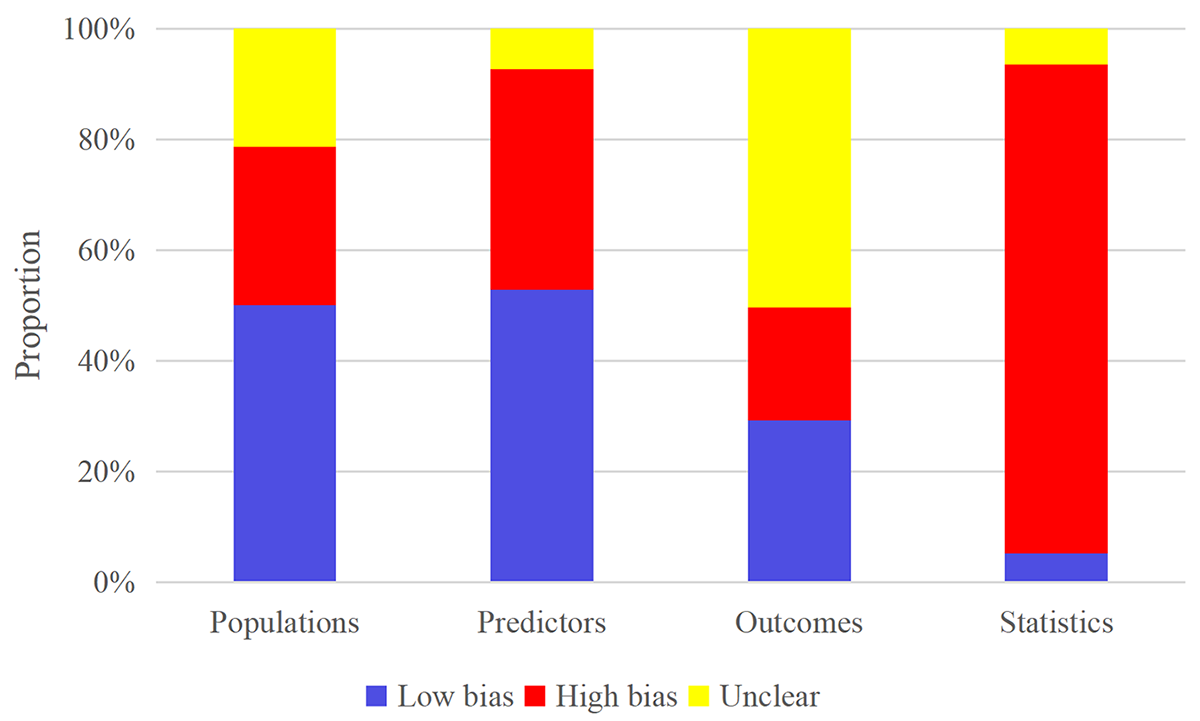

Supplement: Multimedia Appendix 3 [file jmir_v28i1e83821_app3.png]
